# Vaginal microbiome topic modeling of laboring Ugandan women with and without fever

**DOI:** 10.1038/s41522-021-00244-1

**Published:** 2021-09-10

**Authors:** Mercedeh Movassagh, Lisa M. Bebell, Kathy Burgoine, Christine Hehnly, Lijun Zhang, Kim Moran, Kathryn Sheldon, Shamim A. Sinnar, Edith Mbabazi-Kabachelor, Elias Kumbakumba, Joel Bazira, Moses Ochora, Ronnie Mulondo, Brian Kaaya Nsubuga, Andrew D. Weeks, Melissa Gladstone, Peter Olupot-Olupot, Joseph Ngonzi, Drucilla J. Roberts, Frederick A. Meier, Rafael A. Irizarry, James R. Broach, Steven J. Schiff, Joseph N. Paulson

**Affiliations:** 1 Department of Biostatistics, Harvard T.H. Chan School of Public Health and Department of Data Sciences, Dana Farber Cancer Institute, Boston, MA USA; 2 Division of Infectious Diseases, Department of Medicine, Massachusetts General Hospital and Harvard Medical School, Boston, MA USA; 3 Mbale Clinical Research Institute, Mbale Regional Referral Hospital, Plot 29-33 Pallisa Road, P.O. Box 1966, Mbale, Uganda; 4 Department of Biochemistry and Molecular Biology, Institute for Personalized Medicine, Penn State University College of Medicine, Hershey, PA USA; 5 Department of Medicine, Penn State College of Medicine, Hershey, PA USA; 6 CURE Children’s Hospital of Uganda, Mbale, Uganda; 7 Department of Pediatrics and Child Health, Mbarara University of Science and Technology, Mbarara, Uganda; 8 Department of Microbiology, Mbarara University of Science and Technology, Mbarara, Uganda; 9 Department of Women’s and Children’s Health, University of Liverpool and Liverpool Women’s Hospital, members of Liverpool Health Partners, Liverpool, UK; 10 Mbale Clinical Research Institute/Busitema University, 1966 Mbale, Uganda; 11 Faculty of Medicine, Department of Obstetrics and Gynecology, Mbarara University of Science and Technology, Mbarara, Uganda; 12 Department of Pathology, Massachusetts General Hospital, Boston, MA USA; 13 Department of Pathology, Wayne State University School of Medicine, Detroit, MI USA; 14 Center for Neural Engineering and Center for Infectious Disease Dynamics, Departments of Engineering Science and Mechanics, Neurosurgery and Physics, The Pennsylvania State University, University Park, Pennsylvania, PA USA; 15 Department of Data Sciences, Product Development, Genentech, Inc., South San Francisco, CA USA

**Keywords:** Anatomy, Microbiome

## Abstract

The composition of the maternal vaginal microbiome influences the duration of pregnancy, onset of labor, and even neonatal outcomes. Maternal microbiome research in sub-Saharan Africa has focused on non-pregnant and postpartum composition of the vaginal microbiome. Here we aimed to illustrate the relationship between the vaginal microbiome of 99 laboring Ugandan women and intrapartum fever using routine microbiology and 16S ribosomal RNA gene sequencing from two hypervariable regions (V1–V2 and V3–V4). To describe the vaginal microbes associated with vaginal microbial communities, we pursued two approaches: hierarchical clustering methods and a novel Grades of Membership (GoM) modeling approach for vaginal microbiome characterization. Leveraging GoM models, we created a basis composed of a preassigned number of microbial topics whose linear combination optimally represents each patient yielding more comprehensive associations and characterization between maternal clinical features and the microbial communities. Using a random forest model, we showed that by including microbial topic models we improved upon clinical variables to predict maternal fever. Overall, we found a higher prevalence o*f Granulicatella*, *Streptococcus*, *Fusobacterium*, *Anaerococcus*, *Sneathia*, *Clostridium*, *Gemella*, *Mobiluncus*, and *Veillonella* genera in febrile mothers, and higher prevalence of *Lactobacillus* genera (in particular *L. crispatus* and *L. jensenii*), *Acinobacter*, *Aerococcus*, and *Prevotella* species in afebrile mothers. By including clinical variables with microbial topics in this model, we observed young maternal age, fever reported earlier in the pregnancy, longer labor duration, and microbial communities with reduced *Lactobacillus* diversity were associated with intrapartum fever. These results better defined relationships between the presence or absence of intrapartum fever, demographics, peripartum course, and vaginal microbial topics, and expanded our understanding of the impact of the microbiome on maternal and potentially neonatal outcome risk.

## Introduction

The vaginal microbiome consists of an ecological community (CMT) of microorganisms that are important in both maternal and neonatal health^1^. During pregnancy, the vaginal microbiome composition is known to change, which has a role in ascending infection in puerperal sepsis^2^. For neonates, exposure to the vaginal microbiome during birth or through premature rupture of membranes is an important route to early-onset neonatal sepsis^3^. Routine screening and treatment for group B *Streptococcus* (GBS) has reduced the rate of neonatal GBS infection in high-income countries^4^. In sub-Saharan Africa (sSA), group B Streptococcal infections are relatively uncommon in early-onset neonatal sepsis and there is a lack of understanding of how the peripartum vaginal microbiome contributes to maternal and neonatal disease^5,6^. Most maternal microbiome research in sSA has focused mainly on non-pregnant or postpartum composition of the vaginal microbiome^7,8^.

In North America, the composition of the vaginal microbiome in reproductive aged women has been characterized using 16S ribosomal RNA gene (rRNA) sequencing (predominantly V3–V4 hypervariable regions) to determine the presence of culturable and unculturable bacteria in these women^9^. Vaginal profiles were categorized into five (I–V) distinct bacterial CMT state types by hierarchical clustering where patients were assigned a single CMT^9,10^. Subsequent work revealed the temporal dynamics of the bacterial CMTs and a large dynamic shift of CMT composition over a 16-week time period for some patients^7,10^. This highlighted why a single CMT assignment was often not stable over time and the need for a more dynamic representation of a patient’s CMT.

Overall, current studies in African women suggest vaginal CMT compositions distinct from European and North American women^11^. Some of the normal vaginal microbial composition in the sSA specifically possess higher prevalence of *Lactobacillus iners* species and the CMT categorization using hierarchical clustering reports a differentiating number in comparison to that of the western world^11–13^. In addition, CMT associations with HIV status and other diseases such as human papillomavirus and vaginal infections have been described^11,14^. In resource-poor settings in sSA, such as Uganda, there is a higher prevalence of many factors that could potentially affect vaginal microbial diversity, including off-label antibiotic use, sexually transmitted infections including HIV and *Chlamydia*, endemic malaria, and cytomegalovirus (CMV) infections^11,15–17^. Furthermore, these comorbidities impact microbial diversity associated with infant pneumonia, acute diarrhea, sepsis, and postinfectious hydrocephalus^17–19^. Although women living in Africa are at greater risk for malaria, sepsis, and infectious diarrhea, the effect of these infections on the vaginal microbiome has not been characterized^20–23^.

The neonate’s first exposure to microbes is ideally through contact with the maternal vaginal and gut microbiota during the birth process^24,25^. Maternal vaginal CMTs change during pregnancy, thought in part to result from hormonal changes^26^. Higher estrogen levels promote the growth of lactic acid-producing bacteria, shifting the microbiome towards predominantly high-*Lactobacillus* CMTs and increasing diversity postpartum^26,27^. Previous research has also suggested that children born via cesarean delivery compared to those born vaginally are inherently more susceptible to developing autoimmune diseases due to the absence of bacteria such as *Bifidobacterium* in their intestinal microbiome^28,29^. It remains unknown the extent to which the composition of the vaginal microbiome in intrapartum sSA women renders her infant more susceptible to neonatal infection and subsequent sepsis^29^. This is particularly true given GBS neonatal sepsis is uncommon in sSA African women^5,6^.

Most maternal microbiome research in sSA has focused on non-pregnancy or postpartum composition of the vaginal microbiome^7,8^. A recent African study has found an association between preterm birth and increase in microbial diversity during pregnancy, using 16s rRNA-sequencing (rRNA-seq) technique (*n* = 38)^30^. Here we sought to comprehensively define the structure of the vaginal microbiome and its association with maternal fever in 99 laboring intrapartum women in Uganda through 16S rRNA-seq of V1–V2 and V3–V4 hypervariable regions. Given the importance of maternal intrapartum fever as an indicator of infection, we incorporated novel modeling methods to characterize the vaginal microbiomes more fully between febrile and afebrile mothers.

## Results

### Clinical and microbiology characterization

Maternal vaginal samples were obtained from 99 Ugandan intrapartum women during active labor but before birth after obtaining written informed consent (full-term births). Women were enrolled after presenting to hospital in labor for delivery in two districts in Uganda: Mbarara (*n* = 50) in western Uganda and Mbale (*n* = 49) in eastern Uganda (see Supplementary Materials, Online Methods). Various clinical and microbiological features were collected and assessed comparing maternal fever status (Table 1, Supplementary Table 1, Supplementary Table 1, and Supplementary Materials). Overall, from the microbiology culture recovery results, we deduced that the use of laboratory bacteriology technique alone was insufficient, as it would limit us to a certain number of bacteria for investigation, limiting the de novo discovery, and potentially greater sensitivity available with marker-gene high-throughput sequencing technologies (see Supplementary Materials, Methods).

**Table 1:** Overview clinical characterization of febrile vs. afebrile.

Characteristic	Afebrile	Febrile	Odds ratio (95% CI)
Total (*N* = 99)	*n* = 49	*n* = 50	Univariate
Average temperature at recruitment (mean, SD)	36.6 (0.3)	38.3 (0.2)	–
Age in years (median, IQR)	26 (7)	22 (6.3)	0.03 (0.00, 0.26)
HIV infected *n* (%)^a^	5 (10%)	5 (10%)	1.00 (0.26, 3.83)
Parity (mean, SD)	2.8 (1.9)	2.3 (2)	0.61 (0.28,1.32)
Self-reported fever in the last 7 days prior to delivery (%)	4 (6%)	25 (50%)	10.56 (3.56, 39.08)
Self-reported vaginal infection in last 1 month (%)^b^	3 (6%)	3 (10%)	1.70 (0.39, 8.71)
Intrapartum antimicrobial use (%)^c^	2 (4%)	5 (11%)	2.82 (0.57, 20.47)
Hours in labor (median, IQR)	18 (38)	33 (30)	1.19 (0.98,1.46)
Cesarean delivery (%)	11 (22%)	24 (48%)	3.19 (1.36, 7.84)
Malaria RDT or blood smear positive in labor (%)^d^	4 (8%)	16 (32%)	5.05 (1.68, 18.92)
Sample collection site (%)	24 (48%)	25 (50%)	1.04 (0.47, 2.30)
Maternal CMV viral load (%)	16 (32%)	17 (34%)	1.06 (0.45, 2.47)

Odds ratio for hours in labor was estimated for every 10 h. The sample site collection was Mbale vs. Mbarara.

*IQR* interquartile range.

^a^*N* = (49,49).

^b^*N* = (48,49).

^c^*N* = (46,44).

^d^*N* = (47,50).

### Vaginal microbiome CMTs of afebrile and febrile laboring Ugandan women

**Fig. 1:**
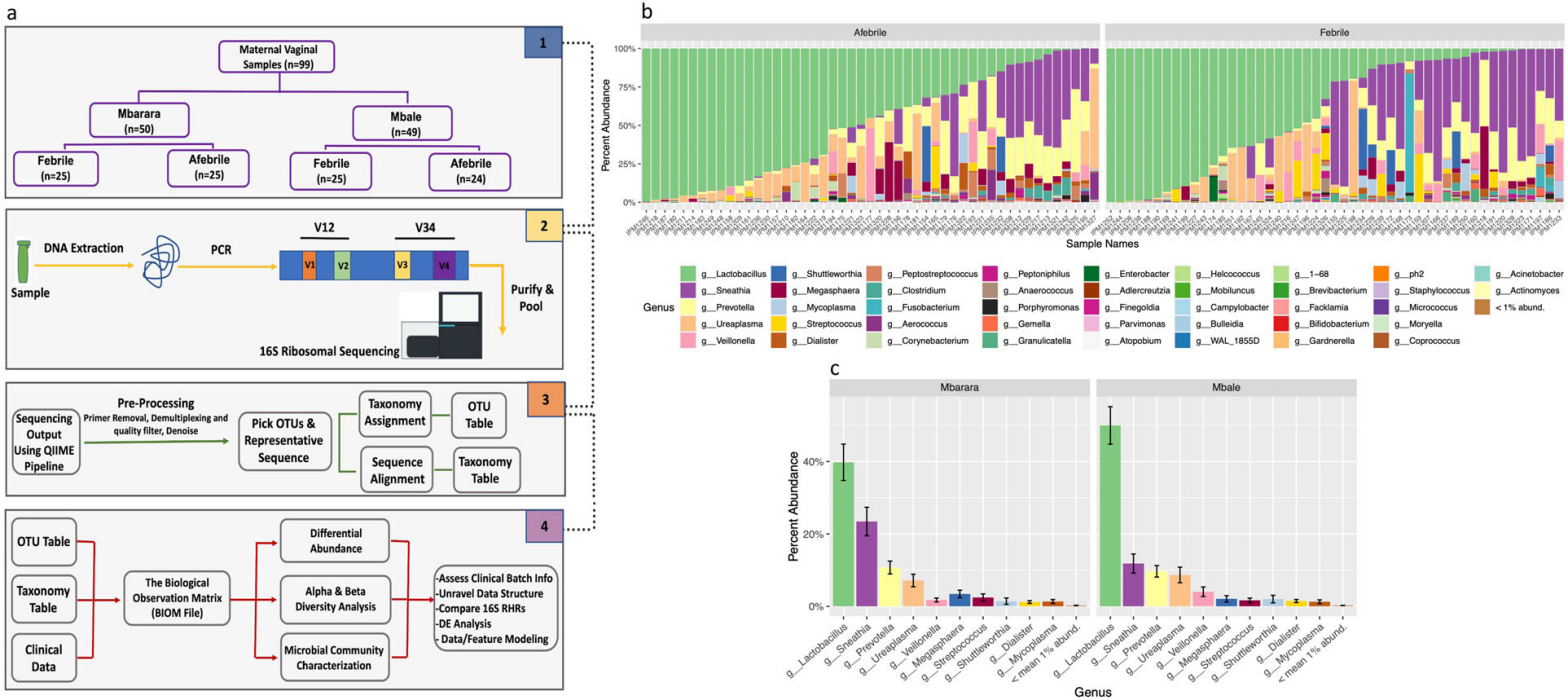
Overall pipeline and structure for 16S ribosomal RNA sequencing (16S rRNA-seq) of maternal vaginal samples. **a** Maternal vaginal samples were collected from two hospital sites in Uganda (Mbarara and Mbale) and were categorized by intrapartum fever status. DNA was extracted and samples underwent library preparation and sequencing on two ribosomal hypervariable regions V1–V2 and V3–V4. The sequence output was pre-processed utilizing the QIIME1 pipeline (“Methods”) and samples were further processed for downstream differential abundance (DA) and modeling in concordance with various clinical and technical variables. **b** Percentage abundance of bacteria on the genus level based on the febrile status of samples. **c** Mean percent abundance of bacteria (agglomerated on the genus level) by enrollment site. (g_) denotes the bacteria naming based on genus taxonomic level.

An overview of our 16S rRNA-seq approach on the V1–V2 and V3–V4 hypervariable regions, and taxonomic assignment method is provided in Fig. 1a, Supplementary Fig. 1, and in the Supplementary Materials. We assessed bacterial recovery in culture and compared it to 16S results (see Supplementary Materials).

We aimed to dissect patterns within our data that could unravel the relationship between vaginal microbiome structure and maternal fever. Through taxonomic assignment and downstream analysis, we estimated the percent abundance of top genera represented in our data set Fig. 1b. As expected, *Lactobacillus* was the most predominant genus in the vaginal microbiome of laboring women^26^. *Sneathia*, often associated with bacterial vaginosis (BV), was the second most predominant genus^11^, followed by *Ureaplasma* and *Prevotella*, all of which are shown to be associated with dysbiosis in preterm birth but also observed as normal members of commensal vaginal flora^31–33^. The distribution of the top genera of bacteria identified was primarily consistent across both sample collection sites Fig. 1c.

We initially examined the vaginal CMTs through previously described hierarchical clustering methods. As there has not been a consensus on the number of CMTs in the vaginal microbiome of African women, we assumed agreement with the number of CMTs expected in the European and African American samples, and picked five as the initial number of clusters to investigate^11^. We pursued two approaches as follows: first the traditional hierarchical approach (see Supplementary Materials) and second a non-dichotomous approach whereby we subsequently performed differential abundance analysis.

Through the second approach, we refined sample clusters on a subset of taxa differentially associated with the traditional hierarchical clustering, to identify CMTs on taxa that could be used as a biomarker in resolving CMT membership and filtering taxa that are not differentially abundant between CMTs (Supplementary Materials). CMT 1 was previously identified as a *Lactobacillus* genera-rich CMT (Supplementary Table 3)^9^. Similarly, previously CMT 2 was mainly composed of high *L. iners* species^9^, whereas CMT 3 appeared to be similar in this regard, but contained a greater abundance of *Finegoldia*, *Veillonella*, and *Streptococcus* genera. CMTs 4 and 5 displayed more diverse CMTs with high levels of *Sneathia*, *Dialister*, *Gemella*, *Clostridium*, and *Prevotella* genera. CMT 5 had higher levels of *Parvimonas*, *Clostridium*, *Mycoplasma*, *Adlercreutzia*, and *Mycoplasma* genera in comparison to CMT 4 (Supplementary Table 4). We additionally identified unique bacterial genera including *Adlercreutzia*, *Granulicatella*, *Bulleidia*, *Staphylococcus*, *Micrococcus*, and *Fusobacterium* in this cohort and CMT (Supplementary Table 3). An association between the maternal CMV viral load and the vaginal microbial CMT was not observed (*P* = 0.57).

**Fig. 2:**
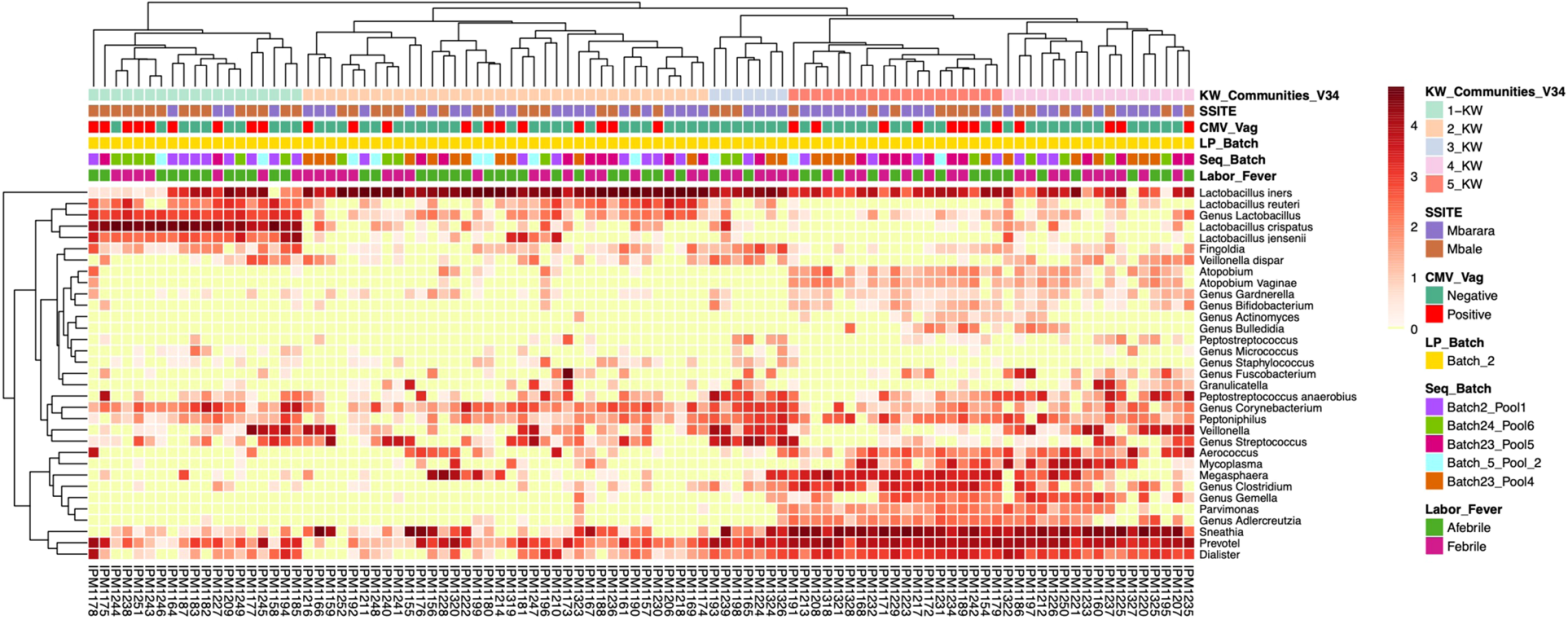
Vaginal bacterial community characterization heatmaps of intrapartum Ugandan women through hierarchical clustering. Vaginal bacterial community classification through selected bacteria after Kruskal–Wallis (KW) test. KW_Communities_V34 are the communities identified by bacteria selected from the KW test. Color of the heatmap represents log10 normalized counts of species and yellow represents zero counts. Annotations are as follows: KW_Communities_V34 is the vaginal community identified from V3–V4 regions through hierarchical clustering followed by bacteria selected by KW test. CMV_Vag represents CMV status of the vaginal samples identified by PCR; LP_Batch is the library preparation batch; Seq_Batch is the sequencing batch, which the samples were processed in; Labor_Fever is the fever status of the laboring woman (see “Methods” for definitions; SSITE is the sample collection site (Mbarara or Mbale).

Furthermore, there was no association between maternal intrapartum fever and CMT classification previously described hierarchical clustering methods (*P* = 0.52) (Fig. 2). When classified using the V1–V2 hypervariable region, similar CMTs were formed; however, this region was not as efficient at speciating *Lactobacillus* and *Streptococcus* species, but identified other unique species compared to the V3–V4 hypervariable region^34^ (Supplementary Fig. 2, Supplementary Table 2, and see Supplementary Materials).

**Fig. 3:**
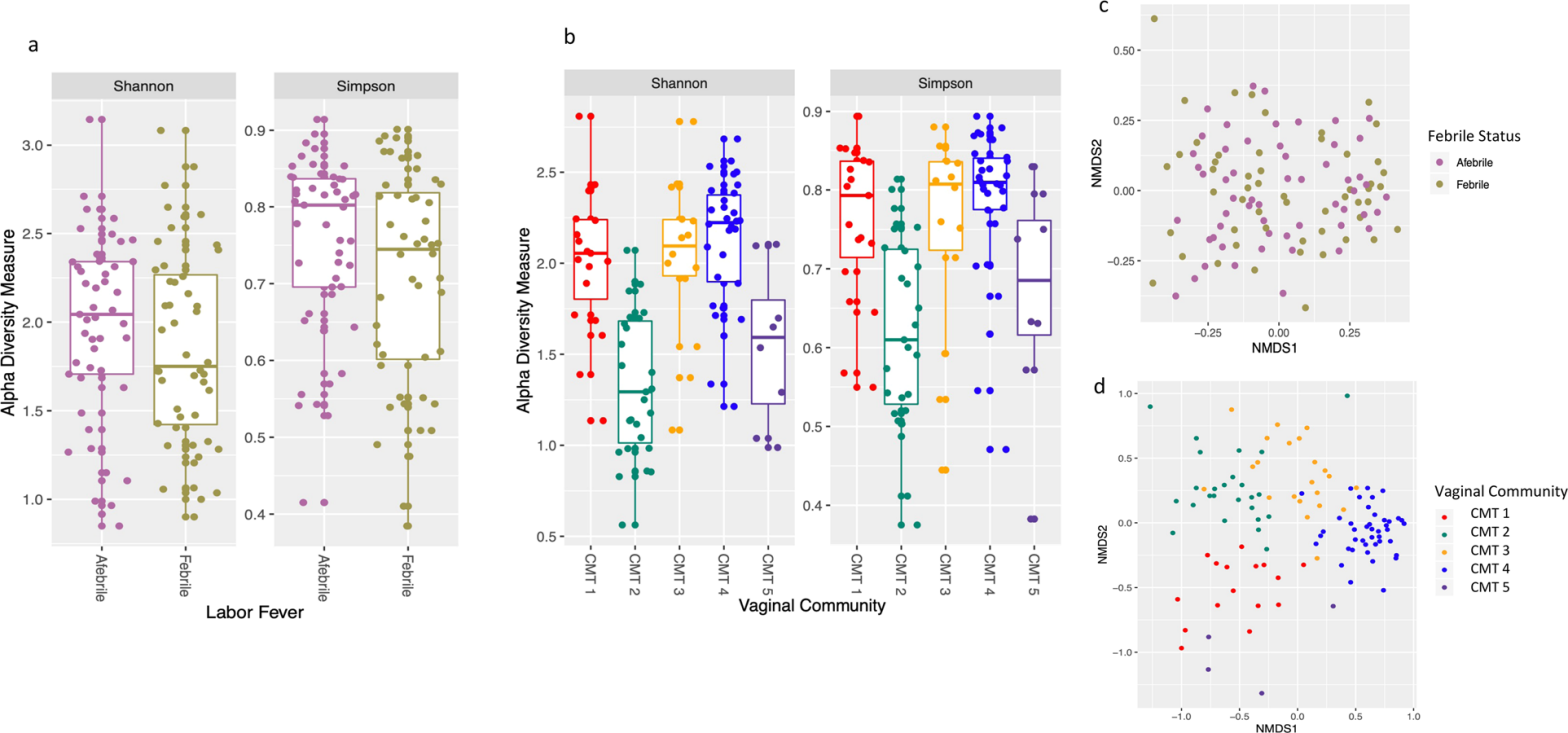
Structure of vaginal microbiome based on diversity estimates. **a** α-Diversity estimation (Shannon, Simpson) jitter boxplot of maternal cohort when fever status is considered utilizing V3–V4 regions (upper and lower quartiles are shown by whiskers and center line represents the median α-diversity). **b** α-Diversity estimation, jitter boxplot of maternal cohort when sample community assignment is taken into account; CMT denotes community (1–5) (upper and lower quartiles are shown by whiskers and center line represents the median α-diversity). **c** β-Diversity of maternal sample cohort shown by non-metric multidimensional scaling (NMDS). Samples are colored based on the maternal fever status (febrile, afebrile), goodness of fit stress = 0.2. **d** β-Diversity of maternal sample cohort by NMDS. Samples are colored by community assignment through hierarchical clustering goodness of fit stress = 0.18.

We assessed α-diversity (Shannon and Simpson)^35^ of the vaginal microbiome comparing fever and CMT states across our samples. We observed no significant difference in α- or β-diversity using either V1–V2 or V3–V4 between febrile states (*P* = 0.25 and *P* = 0.12) (Fig. 3a, c). On the contrary, the microbial CMTs assigned from hierarchical clustering significantly explained the α-diversity (Shannon *P* < 1.78e − 08, Simpson’s *P* < 3.91e − 04) (Fig. 3c, d and Supplementary Fig. 2a–d). Overall, we find that the structure of our sample cohort assessed by α- and β-diversity was highly driven by microbial CMTs assigned by hierarchical clustering and not by maternal fever status. However, although the complexity of the microbiome may not be associated with febrile status, it does not exclude the possibility that individual pathogens are differentially associated with febrility.

### Differential abundance analysis

We tested for associations between bacterial taxa and maternal complications, including fever status. We performed differential abundance analysis for both unadjusted and controlled for CMT and sample collection sites (see Online Methods for detail).

**Fig. 4:**
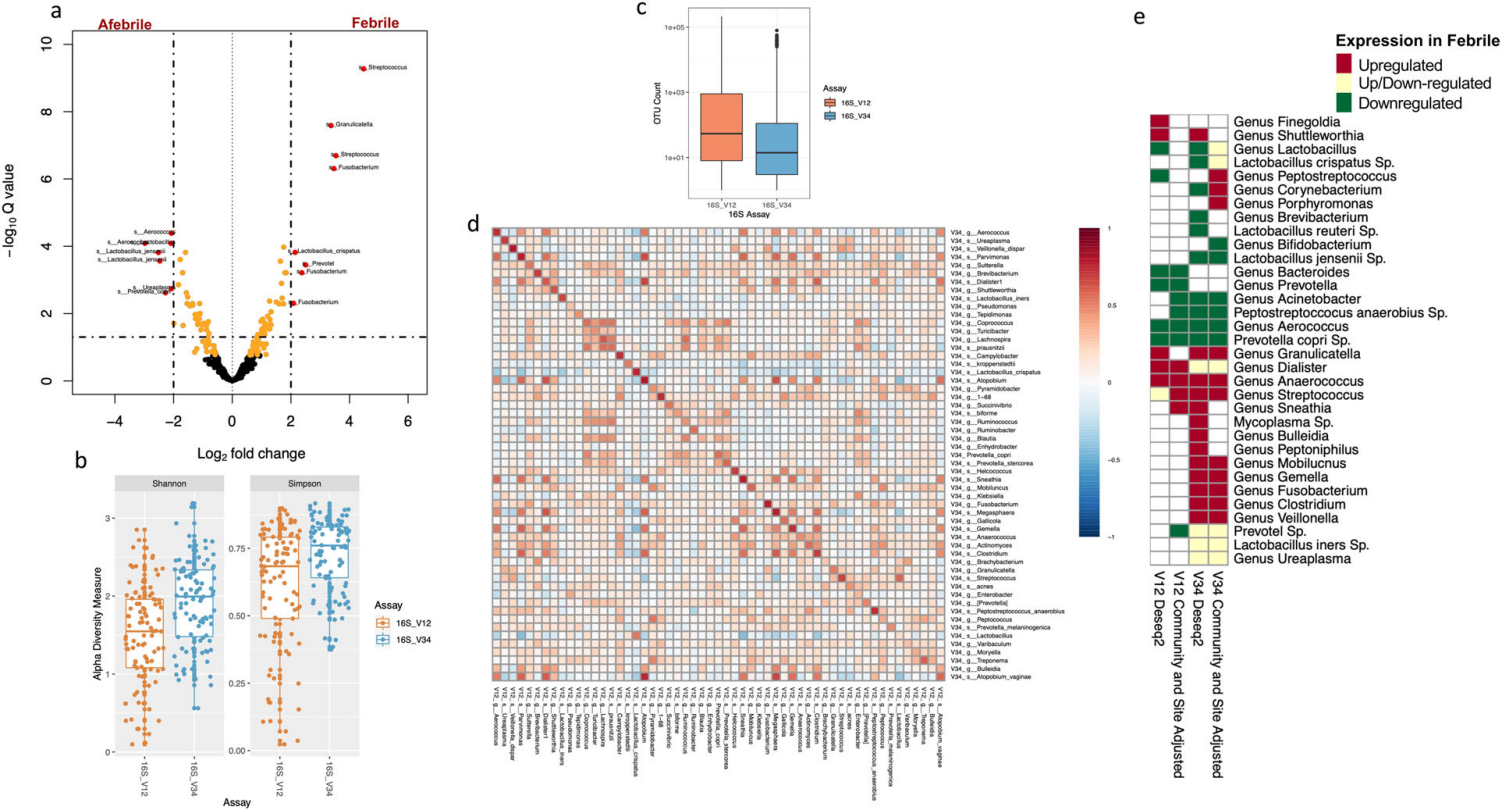
Differential vaginal bacterial presence in febrile vs. afebrile laboring women utilizing V1–V2 and V3–V4 hypervariable regions. **a** Volcano plot representing differentially abundant bacteria in febrile vs. afebrile women using V3–V4 regions. Orange points signify *P* < 0.05 and labeled red points bacterial OTUs with adjusted *P* < 0.05 (Bonferroni correction). s_ represent species level and g_ denotes genus level classification of OTUs. **b** α-Diversity across the V1–V2 vs. V3–V4 assay. **c** Count per OTU in relevance to assay. **d** Spearman’s correlations of samples above zero counts in both V1–V2 and V3–V4 regions (OTUs agglomerated at species level). Spearman’s correlation measures are between (−1,1). **e** Heatmap table of differentially abundant bacterial OTU concordance utilizing V1–V2 and V3–V4 regions from DeSeq2 analysis (*P* < 0.05) (it is noteworthy that the figure is portrayed in increase and decrease in bacteria with and without covariates (site and community assignment CMT) in the Deseq2 model, respectively.

We observed that the abundance of *Granulicatella*, *Streptococcus*, *Fusobacterium*, *Anaerococcus*, *Sneathia*, *Clostridium*, *Gemella*, *Mobiluncus*, and *Veillonella* genera were consistently higher in febrile mothers. We observed *Lactobacillus jensenii, Aerococcus sp.*, *Prevotella copri*, *Acinetobacter sp.*, *Lactobacillus crispatus*, and *Lactobacillus reuteri* at higher abundance levels in afebrile mothers (Fig. 4a, e).

### Reproducibility with differing 16S V1–V2 and V3–V4 hypervariable regions

To assess the consistency of our results between the V1–V2 and V3–V4 hypervariable regions, we compared diversity, read depth, and species abundances across matched samples. In general, α-diversity was lower in V1–V2 relative to V3–V4 (Shannon *P* = 1.41e − 05, Simpson *P* = 1.56e − 4), whereas sample read depth was consistently higher in V1–V2 than in V3–V4 (Mann–Whitney *P* < 2.2e − 16) (Fig. 4b, c).

Species abundances were highly correlated and clustered between the two hypervariable regions (Fig. 4d). In particular, a cluster formed by *Turicibacter*, *Lachnospira*, *Prausnitzii*, and *Coprococcus* (Spearman’s correlation > 0.2). These bacteria are all known members of the gut microbiome family and formed a CMT when measuring correlation across samples. Furthermore, we identified nuanced bacterial relationships between regions. For example, *Mycoplasma sp*. displayed no correlation (Spearman’s correlation = 0.05) across regions; on the other hand, *Dialister* *sp*. had a strong correlation across both regions (Spearman’s correlation = 0.85) (Fig. 4d). We found additional nuances between bacteria taxa depending on the hypervariable region, which we have further explained in the Supplementary Materials.

### GoM models for characterization of vaginal microbial CMTs

Grades of membership (GoM) models have most commonly been used in text processing and document structure identification^36^. One such method employs a Latent Dirichlet Allocation (LDA) approach, a generative latent variable model, where documents can be thought of as bags of words generated by various themes phrased as “topics” and are represented by vectors of frequencies^36,37^. LDA can be used to simultaneously distill topics from a set of “documents” and describe a weight to each document as belonging to a particular topic.

1 $$p\left( {s_i} \right) = \mathop {\sum}\limits_{j = 1}^T {p\left( {s_i|z_i = j} \right)p\left( {z_i = j} \right)}$$

**Fig. 5:**
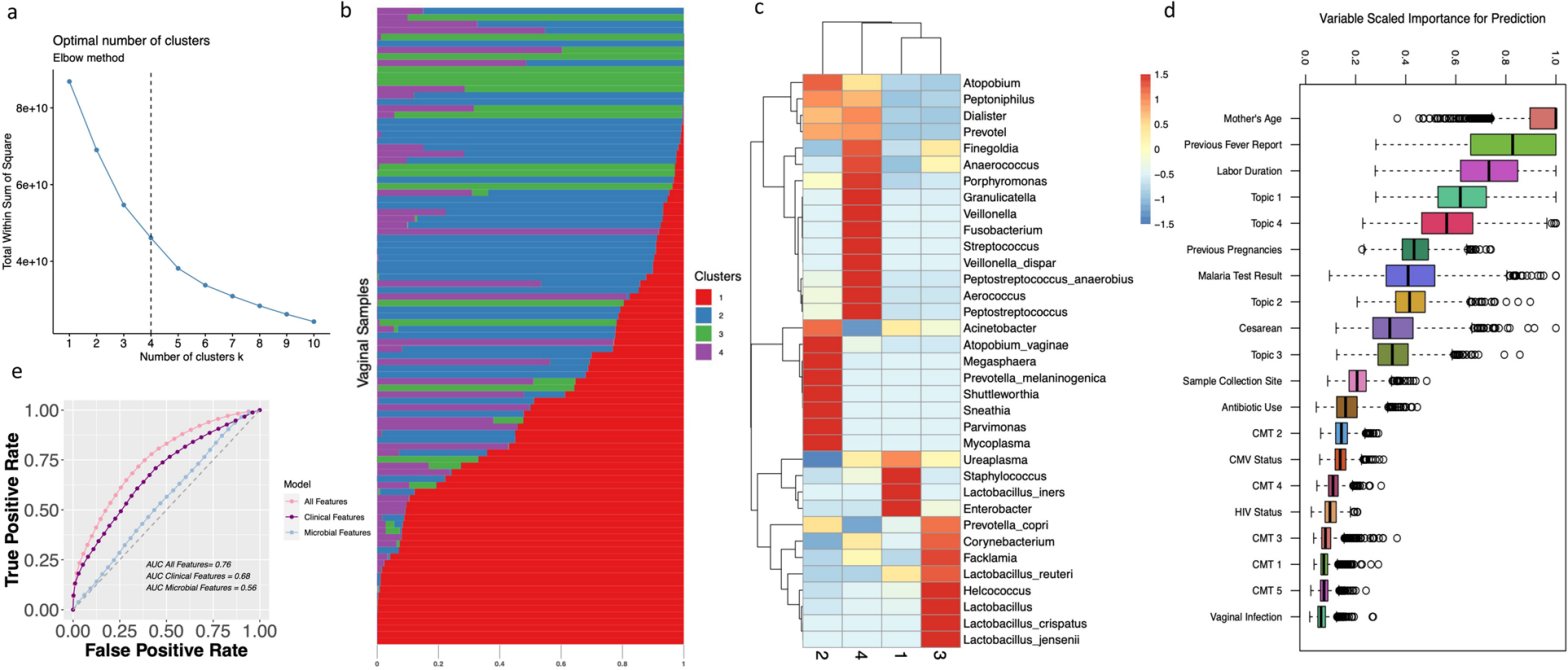
GoM models and random forest for maternal febrile status prediction. **a** Cluster number determination for the data set utilizing the elbow method, which maximized the variance of rate of decline changes as a second derivative. **b** The sample weight value (*ω*) of the topic model for the V3–V4 data set. Every row represents a participant’s vaginal swab sample. Every color represents the ratio in which the sample belongs to that particular cluster determined by the model. **c** Theta (*θ*) value for every feature (on species taxonomic rank) contributing to the formation of the clusters of GoM models. The heatmap score is a row wise *z*-score normalized value for every feature in each cluster. **d** Feature importance for maternal fever status determination utilizing clinical features in addition to topic model clusters and communities identified by hierarchical clustering result of 1000 rounds of random training and test set modeling (CMT 1–5 denote communities identified in hierarchical clustering formatted as binary 1–5 (present absence feature) feature. **e** Receiver operator curve (ROC) for 1000 rounds of random resampling of training and test set of the RF model for maternal fever status identification using all (both clinical and microbial features), clinical, and microbial features.

We applied GoM models to describe the underlying bacterial CMT structures that may have evaded detection in our hierarchical clustering methods, and to quantify the degree to which each sample belongs to the identified topics. Where *T* is the number of topics (bacterial CMTs), preassigned through estimation of optimal numbers of clusters (i.e., topics) to be used for our GoM models via the elbow method and Nonnegative Matrix Factorization^38,39^. Both methods confirmed the optimal number of topics for our data set was four (Fig. 5a and Supplementary Fig. 1a).

Bacterial features were agglomerated at the species level (*s*). We assume *p*(*z*) as the distribution over topics *z* in a particular sample, represented as *ω*, and *p*(*s* | *z*) as the probability distribution over bacterial species (*s*) given topic (*z*). *p* (*z*_*i* = *j*) is the probability that the *j*th topic was sampled for the *i*th bacterial species and *p* (*s*_*i* | *z*_*i* = *j*) as the probability of the *i*th bacterial species (*s*_*i*) under topic *j*, also called *θ* for simplicity. Utilizing the LDA model, we determined the weight of every bacterial species in a topic (*θ*) (1).

The key difference between the GoM models and traditional hierarchical clustering approaches include the ability to no longer rely on a dichotomous representation of a patient’s CMT state profile. In the hierarchical clustering, a patient is explicitly given a CMT from the dendrogram assignment. Leveraging GoM models, we create a basis composed of a small number of microbial topics whose linear combination optimally represents each patient.

We built GoM models to evaluate the sample and bacterial distribution of our data set, and then estimated the topic weights across each sample and topic feature probability distribution, respectively (Fig. 5b and Supplementary Fig. 5b). We discovered a high prevalence of topics 1 and 2 across our sample set. High feature weights were observed for *L. iners*, *Ureaplasma*, *Staphylococcus*, and *Enterobacter* in topic 1. Topic 2 included *Sneathia*, *Shuttleworthia*, *Atopobium*, *Mycoplasma*, *Megasphaera*, and *Parvimonas* consistently, regardless of the hypervariable region used. Topic 3 was enriched in high levels of other *Lactobacillus* species, especially *L. crispatus*, *L. jenesenni*, and *L. reuteri* (Fig. 5c and Supplementary Fig. 5c). Topic 4 was enriched in pathogenic bacterial genera including *Granulicatella*, *Streptococcus*, *Fusobacterium*, V*eillonella*, and *Anaerococcus* (Fig. 5c, Supplementary Fig. 5c, and Supplementary Table 5).

### Clinical associations with GoM-defined topics

Finally, we investigated the relationship between various clinical and environmental features of our data set and the topics identified using our GoM model, and observed intriguing associations. Univariate regression defined interesting associations. Topic 1 was associated with higher odds of both prescribed and non-prescribed antibiotics use (odds ratio (OR) = 9.77, 95% confidence interval (CI) = 1.3–119.11, *P* = 0.04, adjusted *p* = 0.16) (Supplementary Fig. 5d). There was a correlation between increased labor duration and topic 4 (Spearman’s correlation = 0.20, *P* = 0.004, adjusted *P* = 0.01). Intriguingly, topic 3, which is enriched in *Lactobacillus* species, was negatively associated with high labor duration (Spearman’s correlation = −0.19, *P* = 0.03, adjusted *P* = 0.09) (Supplementary Fig. 5d) and topic 4 was weakly associated with higher cesarean delivery incidents (*P* = 0.04, adjusted *P* = 0.16) (Supplementary Fig. 5d). Multivariate regression analysis confirmed a positive association between antibiotic use and topic 1 (*P* = 0.03) (Supplementary Table 5) when adjusting for clinical features (see Online Methods).

### Prediction of maternal febrile status using RF model

To achieve a better understanding of features that may lead to intrapartum fever, we utilized a random forest (RF) classifier model, capable of handling continuous and categorical variables^40^. We used clinical and environmental variables as features (*n* = 11) as well as the weights for each topic and CMTs identified by hierarchical clustering (CMT 1–5) (*n* = 9). We estimated true-positive and false-positive rates by resampling training (*n* = 69) and test data (*n* = 30) from our data set 1000 times. Maternal age was the most important feature predicting the presence or absence of intrapartum fever, followed by maternal reports of previous fever during pregnancy. Interestingly, topic 1, labor duration, and topics 2–4 were important correlates of maternal fever status (Fig. 5d and Supplementary Fig. 6b, d). The performance of the model was examined using receiver operator curves and the area under the curve (AUC) was estimated (AUC = 0.76) (Fig. 5e and Supplementary Fig. 6a, b). In addition, we measured the model performance for clinical and microbial features individually, and found a decrease in performance when compared to the model incorporating both features (AUC = 0.68 and AUC = 0.56, respectively) (Fig. 5e and Supplementary Fig. 6b, c).

## Discussion

Heterogeneity in the vaginal microbiome exists due to environmental exposure, behavioral factors, genetic variability, or medical comorbidities, and may differ between continents and across populations^41–43^. Changes in the vaginal microbial CMT could lead to increased risk of early-onset neonatal sepsis due to the presence of pathogenic bacteria or dysbiotic CMTs^42,44^. The association between the vaginal microbiome and maternal fever during labor has been incompletely studied. Here we characterized the vaginal microbiome in laboring Ugandan women and found associations with intrapartum fever. We demonstrated that novel microbial topic models can be useful for predicting maternal fever. The GoM modeling approach was a necessary probabilistic method to extend the dichotomous representation of a patient’s CMT state profile with greater use in the recent microbiome literature^45–47^. Our improved results construct a framework for future validation studies based on a more ecological characterization of the microbiome and its potential pathogenic effects.

A comparison with standard microbiology vs. 16S rRNA-seq (V1–V2 and V3–V4) showed a greater sensitivity and broader bacterial capture capability using 16S rRNA-seq. Using two different hypervariable regions, V1–V2 and V3–V4, led to a more comprehensive vaginal microbiome analysis. Although commonly identified taxa were often correlated, there were unique taxa identified by each of the two regions.

We characterized the vaginal microbiome through widely used supervised and unsupervised hierarchical clustering to identify bacteria unique to vaginal CMTs. As previously reported, using supervised clustering we found higher abundance of *L. iners* species in our African CMT 2 and CMT 3 populations (Supplementary Table 4)^11^; in addition, we found higher overall diversity of bacteria across all CMTs compared to unsupervised approaches. The bacteria previously associated with BV (CMT IV as defined by Ravel et al.^9^)were both more abundant and present in more CMTs (CMT 2, CMT 4, and CMT 5) within our cohort.

Through differential abundance analysis comparing febrile vs. afebrile laboring women, we found that bacterial genera *Granulicatella* (a nutritionally variant bacteria, which has been associated with a variety of infectious pathogens), *Sneathia*, *Streptococcus*, *Fusobacterium*, *Clostridium* (four known BV associated bacteria), Anaerococcus, and *Veillonella* (both commensal bacteria) were consistently more abundant in febrile women. Some of these genera have previously been associated with bacterial vaginosis and are known pathogens^48–53^. However, to our knowledge, no explicit association has been described between these bacteria and maternal intrapartum fever^9^. The Gram-negative bacterial genus *Sneathia* has been associated with high levels of inflammation and the presence of vaginal proinflammatory cytokines interleukin (IL)-1α, IL-1β, and IL-8 proteins^54^. High levels of GBS (*Agalactiae* *sp*.) have been described as a common cause for neonatal sepsis^55^. Although we were unable to speciate this microbe utilizing V1–V2 primers, we were able to speciate and detect this bacteria in 29% of both febrile and afebrile patients by V3–V4 primers or bacterial recovery cultures^55^. Previous research on GBS prevalence in pregnant women reported a 17%, 6–36%, and 29% rate of infection in Guatemala, Denmark, and the United States, respectively^56,57^*. L. jensenii*, *Aerococcus sp.*, *Acinetobacter sp.*, *P. copri*, *L. crispatus*, and *L. reuteri* were more abundant in afebrile women.

As we could not identify any significant association between previously described vaginal microbiome CMTs through previously described hierarchical clustering methods, we used the novel GoM approach for vaginal microbiome CMT identification. Leveraging the application of GoM topic models, we were able to better characterize and understand the underlying structure of the antepartum vaginal microbiome. This approach enabled us to determine the best topics that fit for each individual in the cohort rather than restricting each individual to a specific CMT. Through this method, we identified four topics (1–4) prevalent in the cohort.

We identified a number of associations between the identified topics and clinical characteristics. Maternal antibiotic use was associated with topic 1 (OR = 9.77 (95% CI: 1.3–119.11)) and was enriched in *L. iners*, *Ureaplasma*, *Staphylococcus*, and *Enterobacter* genera. This association has implications for the disruption in vaginal flora that may be caused by antibiotic use, whether prescribed or self-administered. *L. iners* has been shown to be the most adaptable, and resilient to fluctuations in the vaginal nitch^58^, in this case, to changes potentially due to antibiotic use. Recent studies have also found dominance of vaginal bacterial CMTs with *L. iners* is a risk factor for preterm birth^59^. Furthermore, we found that decreased labor duration was associated with topic 3, *L. jensenii*, *L. crispatus*, *Acinetobacter sp*., and *L. reuteri* (Supplementary Fig. 7 and Supplementary Table 6). Finally, topic 4 manifested weak associations with higher cesarean delivery incidents. In contrast, topic 4 was associated with longer labor duration and cesarean delivery. Specific bacteria in topic 4 were shown to be more prevalent in febrile mothers, including *Streptococcus sp*., *Granulicatella sp.*, and *Veillonella* *sp*. Our findings demonstrate the value of GoM topic models to categorize the structure of the vaginal microbial CMT in laboring women.

Finally, we fused CMT, topics, and clinical features in our data set into an RF model. Although age has been previously described as associated with febrility, we found that young maternal age was the strongest factor in predicting fever. Surprisingly, topic models were shown to be among the dominant features, affirming the GoM topic approach to microbial CMT structure. Topic 1—*L. iners*, *Ureaplasma*, *Staphylococcus*, and *Enterobacter*—was the fourth most important feature for determining maternal febrile status followed by topic 4. Topic 4 was more prevalent in febrile mothers consistent with the presence of *Sneathia*, *Granulicatella*, *Anearococcus*, and *Streptococcus* *sp.* in febrile women, and the fifth most important feature in determining maternal health. The RF model also demonstrated that all four topics describing the vaginal microbiome CMTs of intrapartum women showed importance in predicting intrapartum fever classification in the model, suggesting consequence of combinatory presence of bacteria in individual mothers; this is in contrast with the traditionally employed CMTs defined through hierarchical clustering.

Our findings support, using a more multifaceted methodological approach, leveraging multiple models to characterize such microbiomes across populations. In the case of the vaginal microbiome, this more comprehensive approach identifies structural microbiome features that better predict maternal health and risk for intrapartum women and their neonates. Recent studies in Nigerian cohorts (shown through Nugent score, Gram staining, and 16s rRNA-seq) have linked more diverse microbiome composition during pregnancy to low birth weight and preterm membrane rupture; hence, applying these multifaceted approaches in order to identify potential links and risks in neonatal outcome is crucial^12,30^.

We found a higher prevalence o*f Granulicatella*, *Streptococcus*, *Fusobacterium*, *Anaerococcus*, *Sneathia*, *Clostridium*, *Gemella*, *Mobiluncus*, and *Veillonella* genera in febrile women, and higher prevalence of *Lactobacillus* genera, *Acinetobacter*, *Aerococcus*, and *Prevotella* species in afebrile women. We show by integrating clinical variables with microbial topics into this model that young maternal age, fever reported earlier in the current pregnancy, and longer labor duration, as well as a more diverse, less *Lactobacillus*-dominated microbiome were features of labor associated with intrapartum fever.

This small exploratory study with 99 participants demonstrates that more sophisticated strategies to understand the maternal microbiome may be important in understanding which combinations of genera may contribute to febrile illness in mothers. Future studies will require larger sample sizes to help further characterize this complex microbiome and its interaction with maternal and fetal health.

## Methods

### Eligibility criteria, ethical approval, and consent to participate

This study protocol was approved by the institutional review boards at each participating institution, including Mbarara University of Science and Technology (MUST) Research Ethics Committee (12/11-15), Mbale Regional Referral Hospital Research Ethics Committee (082/2016), Uganda National Council of Science and Technology (HS/1963), Partners (2016P000806/PHS), and Pennsylvania State University College of Medicine (STUDY0004199). Participants were recruited from two hospital sites (Mbarara and Mbale) in Uganda. An equal number of participants were recruited from Mbarara Regional Referral Hospital (MbararaH) in Mbarara, Uganda, and Mbale Regional Referral Hospital in Mbale, Uganda (MbaleH). MbararaH is an ~300-bed academic hospital affiliated with Mbarara University of Science and Technology, with 9000 deliveries annually. MbaleH is a 400-bed public hospital that has nearly 10,000 deliveries a year. Both hospitals serve a mixed urban-agrarian population.

Materials Transfer Agreement was in place between MUST and Pennsylvania State University. An Institutional Biosafety Committee provided oversight of specimen handling at Penn State. Maternal oral temperatures were measured during labor using a digital thermometer (ADC ADTEMP Hypothermia Digital Thermometer), to confirm eligibility.

Verbal consent was given by mothers of at least 18 years of age. These mothers delivered at term (≥37 weeks’ gestation) and had an intrapartum oral temperature measurement between 36.0 °C and 37.5 °C (afebrile group, *n* = 25 per site), or an intrapartum oral temperature measurement >38.1 °C on one occasion or >38.0 °C twice, at least 60 min apart (febrile group, *n* = 25 per site). Maternal peripheral blood and vaginal swabs were taken at the time of labor and clinical data were collected.

### Sample collection

After informed consent was obtained, maternal peripheral blood and vaginal samples were obtained. A maternal blood sample was collected using aseptic technique. Rapid diagnostics and a thick and thin blood smear were prepared for malaria testing (SD Bioline Malaria Ag P.f/Pan). Two maternal vaginal swabs were collected consecutively during labor. First, a sterile DNA- and RNA-free swabs for 16S rRNA-seq was inserted into the posterior vaginal fornix after gently cleansing the perineum with clean gauze. The swab rotated for 2–3 s, inserted into a preservative-filled (DNA/RNA Shield, Zymo Corporation) cryovial, and vortexed at high speed for 10 s. Swabs were then frozen in liquid nitrogen and stored at −80 °C prior to cryogenic transfer to the United States for sequencing. A second swab for microbiologic culture (BD BBL CultureSwab Plus Amies Gel) was then inserted into the posterior vaginal fornix immediately following collection of the first swab, rotated for 2–3 s, and placed back into the media-containing swab container. Culture swabs collected at Mbarara Hospital (MbararaH) were transferred to the on-site microbiology laboratory within 36 h, whereas those collected at Mbale Hospital (MbaleH) were 4 °C for up to 48 h before transport to a commercial microbiology laboratory in the Ugandan capital Kampala by road. Clinical data were obtained from structured interviews and chart review collected before and after delivery.

### Microbiology

Vaginal swab samples collected from MbararaH were processed in the adjacent MUST microbiology lab. A Gram stain was prepared and swabs were plated on MacConkey agar, incubated at 37 °C, and checked for daily growth. When growth was observed, colonies were plated onto various selective and nonselective growth media and re-incubated. Following incubation, each colony type was enumerated, isolated, and identified using standard biochemical methods. Vaginal swab samples collected from MbaleH were processed at MBN Clinical Laboratories in Kampala. Interpretation of the MbararaH microbiology was performed by a single microbiologist and interpretation of MbaleH microbiology was performed by multiple staff microbiologists.

### Cytomegalovirus PCR reaction

TaqMan PCR assay targeting the CMV *UL54* gene was utilized using region-specific primers and probes: CPOL-F720: 5′-GCTGACGCGTTTGGTCATC-3′, CPOL-R780: 5′-ACGATTCAC GGAGCACCAG-3′, and CPOL-741FAM: 5′-TCGGCGGATCACCACGTTCG-3′. PCR was performed based on recommendations by Habbal et al.^60,61^. In brief, amplification was done on the ABI^TM^ QuantStudio 12K Real-Time PCR Instrument (USA, CA) with the following cycling conditions and times: 60 °C for 30 s, 95 °C for 5 min, then 45 cycles of 95 °C for 15 s, and 60 °C for 1 min. CMV-positive condition was considered for samples with DNA amplification (*C*_t_ < 45) with technical duplicates, and in cases of inconsistency a triplicate was considered. Standard curve analysis was done for all PCR runs, overall efficiency was >75%, and *R*^2^ was >0.95 for all runs (Supplementary Table 7).

### Sample extraction, library preparation, and sequencing

Vaginal specimens were collected in 1 mL of DNA/RNA Shield. After collection, 1 mL of specimen was added, with swab, to 0.15 mm and 0.5 mm zirconium oxide beads, and processed in a Bullet Blender (Next Advance, NY, USA) at high speed for 5 min. After lysis, DNA was extracted from 500 μL of the homogenized sample using ZymoBIOMICS DNA Miniprep Kit (Zymo, CA, USA) following the manufacturer’s protocol with proteinase K digestion and was eluted two times with 50 μL of heated elution buffer. Primer extension PCR reaction of the 16S rRNA variable regions V1–2 and V3–4 was done to reduce reagent contamination using region-specific primers. Primer sequences are as followed: 27F: 5′-AGAGTTTGATCMTGGCTCAG-3′, M13: 5′-CAGGGTTTTCCCAGTCACGAC-3′, 341F_M13: 5′-CAGGGTTTTCCCAGTCACGACCCTACGGGNGGCWGCAG-3′, and 805R: 5′-GACTACHVGGGTATCTAATCC-3′^62,63^. Specifically, for the V1–2 region, the annealing probe was the 336R primer attached to M13 (336R_M13) and then the extended product was amplified with 27F as the downstream bacterial primer and M13. For the V3–4 region, the annealing probe was the 341F primer combined with M13 (341F_M13) and the extended product was amplified with the 805R primer and M13. Amplification was done with 500 nM primers with the MolTaq 16S Mastermix (Molzym GmbH & Co Kg, Germany)^64^. For library preparation, after the amplified products were put into a 1× AMpure XP (Beckman Coulter) clean up, the Hyper Prep Kit (KAPA Biosystems, USA) library preparation kit was used following the manufacturer’s protocols with seven cycles of library amplification. Library was quantified with Qubit or Agilent Bioanalyzer DNA 1000 Chip, and sequenced on Illumina’s Mi-Seq system using the 600 cycle v3 kit with 20% PhiX and 6pM pooled library. Sequencing was performed in Penn State College of Medicine Genomic core (Supplementary Table 7).

### Sequence alignment and taxonomic assignment

Paired-end sequences were filtered for quality using Trimmomatic (version (v) 0.38)^65^. Sequences <100 base pairs (bp) in length and average quality score < 30 on a window of 20 bp were discarded. The remaining paired-end sequences were then joined utilizing PEAR v 0.9.6)^66^. Only joined sequences with designed primers and length > 260 bp were kept. After chimeras were identified and removed using USEARCH method^67^, sequences were clustered into operational taxonomic units (OTUs) via QIIME packages (v 1.9.1)^68^. Sequences of over 97% identity represented the same genus/species and were clustered into the same OTU, and were assigned a taxonomy by Greengenes database (v 13.8). Those OTUs without taxonomy assignment were further blasted with BlastX (v 2.7.1) to non-redundant proteins databases.

### Computational pipelines and statistical methods for downstream analysis

The biological observation matrix object compromising OTU file, phenotype data, and taxonomic assignment file was built using phyloseq and metagenomeSeq packages in the R programming language^69,70^. Minimum inclusion criteria for sequenced samples were 1000 reads. Taxa with less than two reads in 10% of samples were excluded from the analysis. The number of OTUs after filtering using V1–V2 primers was 274 and utilizing V3–V4 primers was 401 OTUs. For the hypervariable region/primer comparison section, we relaxed the filtering criteria to taxa with fewer than one read in 5% samples, to ensure accurate estimates of the presence and absence of association tests for bacteria across both regions.

### Packages used for computational analysis

β-Diversity was estimated using non-metric multidimensional scaling (NMDS). Euclidean distance was measured for hierarchical clustering using the pheatmap package (v 1.0.12). Phyloseq and ggplot2 were used to estimate α (Shannon and Simpson) and β (NMDS) diversity and visualization of plots accordingly^69,71^. Both adjusted and unadjusted differential abundance analyses were performed using DESeq2^72^. Multivariate models were adjusted for microbial CMT and site of collection. GoM models identified were obtained using the CountClust package^73^. Univariate and multivariate regressions were performed for topic weights regressing on various clinical features depending on the type of regression and adjusted by the number of models (i.e., based on the number of topics). RF models were utilized for maternal fever status prediction from the h2o package (v 3.30.0.1, https://github.com/h2oai/h2o-3).

### Statistical tests

All statistical tests and regression analyses were performed using R base functions or MASS^74^. All *p*-values were adjusted using Bonferroni (significance cutoff, *p* < 0.05, and Bonferonni *p* < 0.1) multiple comparison test method R v 3.6.2.

### Reporting summary

Further information on research design is available in the Nature Research Reporting Summary linked to this article.

## Supplementary information

Supplementary Material

Reporting Summary

## Data Availability

The 16S rRNA-sequencing files and metadata can be accessed through https://microbiomedb.org/ and NCBI SRA under BioProject accession #PRJNA672786.

## Code availability

All scripts and statistical methodology can be found in https://github.com/DataScienceGenomics/Vaginal_Microbiome.git.
