# Supplementary material for: Vaginal microbiome topic modeling of laboring Ugandan women with and without fever
Source: NPJ Biofilms Microbiomes. 2021 Sep 10;7:75. doi: 10.1038/s41522-021-00244-1 (PMC8433417; doi:10.1038/s41522-021-00244-1)
Supplement: Supplementary file 1 — Supplementary Material [file 41522_2021_244_MOESM1_ESM.pdf]

Supplementary Material for
Vaginal Microbiome of Peripartum Women in Uganda

Maternal clinical feature assessment

Peripheral blood and vaginal samples were collected 2-120 hours before delivery. Blood samples were tested for cytomegalovirus (CMV) using polymerase chain reaction (PCR) assay and malaria status through both rapid diagnostic test (SD Bioline Malaria Ag P.f/Pan) and traditional blood smear assessment using Giemsa-stained thick and thin smears. Various environmental, health and socioeconomic metadata were collected from these mothers (For more information see Online Methods). Maternal age, delivery mode (Caesarean versus vaginal delivery), malaria infection, and labor duration were each potentially associated with maternal fever Table 1 and Supplementary Table 1.

Overview of 16S ribosomal sequencing

The total number of mapped reads to amplicon sequences after filtering (see methods for detailed filtering criteria) was 13,923,043 for V1-V2 and 5,074,718 for V3-V4 with a median per sample of 138,901 for V1-V2 and 57,048 for V3-V4 . Total number of taxa observed across samples was 277 was V1-V2 and 404 using V3-V4 primers. Significant batch differences were not present in various steps of DNA extraction, library preparation or sequencing Supplemental Figure 1.

Assessment of bacterial cultures and 16S amplicon sequencing

To assess bacterial recovery in culture as compared to 16S assays, the samples were further evaluated in a microbiology laboratory for particular pathogenic bacteria *Klebsiella*, *Staphylococcus aureus (Staph. aureus), Streptococcus agalactiae (Strep. agalactiae),* and *Corynebacteria*. Assuming microbiology recovery as the gold standard, we evaluated the specificity (probability of 16 S negative results while culture is positive), sensitivity (percent of positive 16S results when the culture is positive), and precision (positive predictive value) of 16S methods for both V1-V2 and V3-V4 primers Supplemental Table 2. Depending on the bacteria, we observed overall higher specificity (range 0.06-1, median = 0.72) and moderate sensitivity (range 0.00-1.00, median = 0.57) and low precision (range 0.00 - 0.50, median = 0.05). Additionally, the estimated Cohen's Kappa coefficient of agreement between bacterial recovery culture and V3-V4 for *Klebsiella, Staphylococcus aureus (Staph. aureus), Streptococcus agalactiae (Strep. agalactiae),* and *Corynebacteria* were estimated as 0.32, -0.08, -0.06, and 0.5 and for V1-V2 0.21, NA, NA, 0.01 respectively. We observed the 16S V3-V4 primers could speciate the *Strep. agalactiae*; however, 16S V1-V2 primers could identify only up to the genera taxonomic rank for the latter bacteria. We conclude that use of laboratory microbiology technique alone for this study was insufficient as it would limit our discoveries to a certain number of bacteria for investigation, this would limit de novo

discovery, and potentially greater sensitivity available with marker-gene high-throughput sequencing technologies.

Hierarchical clustering of maternal vaginal microbiome through previously described bacteria

To unravel the structure of the peripartum microbiome in our samples, we initially examined the vaginal communities through previously described hierarchical clustering methods. Since there has not been a full consensus on the number of communities expected in the vaginal microbiome of the African samples, we assumed agreement with the number of communities in the European and American sample and picked five as the initial number of clusters to be investigated. Hence, we pursued two approaches; first, we only considered the previously described genera which were described as forming the vaginal communities in the literature which is described in Supplemental Fig. 2a. The next approach was the non-dichotomous approach described in the main text. As expected, the bacterial community description was altered in comparison to the initial literature on vaginal microbiome content. In contrast to the four distinct communities driven by *Lactobacillus* species in the vaginal microbiomes of the European and American samples, we observed only one *Lactobacillus* rich community containing all species of this genera in our sample set (community (CMT) 1) (1, 2). Additionally, the sample set exhibited a high level of *L. reuteri* in this community, which was not observed in previous vaginal microbiome characterization studies. Although CMT 2 in our sample set had the highest levels of *L. iners* (Kruskal-Wallis adjusted p value (KW p value)= 2.29e-08) across the communities -- consistent with previous literature in the vaginal microbiome of African women -- *L. iners* was prevalent across the sample set (3). CMT 3 was formed by high levels of *Veillonella* sp. in addition to the *Streptococcus* genus, and this community had similar composition to the BV community previously described in the Tanzanian study(4). CMT 4 constituted lower levels of *Veillonella sp.* in addition to the *Streptococcus* genus and higher prevalence of *Parvimonas, Mycoplasma, Gardenerella, Atopobium, Aerococcus, Shuttleworthia, Megasphaera*, and *Clostridium* Genus. CMT 5 appeared to contain high levels of *L. iners* in addition to high levels of *Sneathia, Streptococcus* and *Veillonella* sp. We could not identify any significant correlation between the maternal status of the mother or sequencing batch and the vaginal communities detected through this analysis (Bonferroni adjusted p value = 1). We could, however, identify a positive trend between CMT 1 and maternal CMV status, although this association was not significant after multivariate correction (P = 0.06). Additionally, we looked at correlations between sites of the sample collection and the communities observed, observing again a positive trend with CMT 1 (p value = 0.05) (higher in Mbale than Mbarara). Overall, we favor the blind approach of community characterization for previously under-investigated populations, as it could provide identification of new bacteria genera representation in the vaginal communities Fig. 2. Finally, while we did not observe an association between maternal CMV status and community classification using our hierarchical clustering approach on all taxa, we did observe positive correlations between CMV

viremia and CMT1 and 3 using hierarchical clustering on the subset of species reported by Ravel, et al (1).

16S ribosomal sequencing primers on V1-V2 and V3-V4

Additionally, to better dissect the uniqueness of the two primers at hand, we selected on the species-level OTUs exclusive to either V1-V2 or V3-V4 primers. In our dataset, V3-V4 primers were better able to speciate *Staphylococcus* and *Streptococcus* bacteria than V1-V2 primers (Supplementary Fig. 4b). *Similar to previous reports we also confirmed that Lactobacillus jensenii was identified with V3-V4 primers, but not in V1-V2 primers (5). Unique taxa identified by V1-V2 primers and not V3-V4 hypervariable regions include the observation of an infrequent, but normal* ***Lactobacillus Vaginalis*** *species and* ***Neisseria sp.*** *observed in the V1-V2 region (6).* We observed a high correlation (Spearman correlation = 0.73) between *L. reuteri* and *Phascolarctobacterium* which, although quite distinct taxa, are observed concordantly as abundant in our dataset (Supplementary Fig. 4c).

By utilizing 16S ribosomal sequencing primers on V1-V2 and V3-V4 regions we identified particular species which are differentially expressed between afebrile and febrile mothers. These species include, but are not limited to, *Anaerococcus, Granulicatella, Sneathia, Streptococcus, Lactobacillus jensenii, Aerococcus, Prevotella copri, Acinetobacter, and Peptostreptococcus anaerobius* (Fig. 4e, and Supplemental Fig. 4d). Thus, comparing results from two different hypervariable region primers in 16S sequencing can serve not only to confirm taxa detected, but also unravel nuanced relationships between taxa which can identify new bacteria.

Supplemental Figure Legend

Supplemental Fig. 1: Batch processing of maternal vaginal samples.

a. DNA Extraction batch for both V1-V2 and V3-V4 primers. Abscissa represents the date of extraction. Ordinate denotes library abundance (counts) of OTUs per extraction date. **b.** Library preparation batches for V1-V2 and V3-V4. **c.** Sequencing pool batch abundance for V1-V2 and V3-V4. **d.** Sequencing batch abundance dates for processing of V1-V2 and V3-V4.

Supplemental Fig. 2: V1-V2 Vaginal bacterial community characterization heatmaps

a. Vaginal bacterial community identification through hierarchical clustering of previously identified bacteria V3-4. Color of the heatmap represents log10 normalized counts of species, yellow represents zero counts. Annotations: Vaginal_community is the vaginal community identified from V1-2 and V3-V4 primers through hierarchical clustering. CMV_Vag, represents CMV status of the vaginal samples identified by PCR, LP_Batch is the library preparation batch, Seq_Batch is the sequencing batch which the samples were processed in, Labor_Fever is the febrile status of mother's (Afebrile 36˚ < temp < 37.5˚, Febrile (temp>38˚), SSITE is the sample collection site (Mbarara, Mbale). **b.** Vaginal bacterial community identification through hierarchical clustering of previously identified bacteria V1-2. **c.**Vaginal bacterial community classification through selected bacteria after Kruskal-Wallis test. KW_Communities are the communities identified by bacteria selected from the KW test.

Supplemental Fig. 3: Alpha and beta diversity estimated for V1-V2 16S sequencing

a. Alpha diversity estimation (Shannon, Simpson) jitter boxplot of maternal cohort when febrile status is taken into account utilizing V3-V4 primers (Kruskal-Wallis Shannon and Simpson P = 0.49 and 0.51 accordingly). **b.** Alpha diversity estimation (Shannon, Simpson) jitter boxplot of maternal cohort when samples when sample community assignment is taken into account; CMT denotes community (1-5) (KW Shannon and Simpson P = 1.001e-08, 3.177e-07 accordingly). **c.** Beta diversity of maternal sample cohort shown by nonmetric multidimensional scaling (NMDS). Samples are colored based on the maternal febrile status (febrile, afebrile). **d.** Beta diversity of maternal sample cohort by NMDS. Samples are colored by community assignment through hierarchical clustering.

Supplemental Fig. 4: Expression profile of febrile versus afebrile laboring women utilizing V1-V2 and V3-V4 primers.

a. Volcano plot of differentially expressed species in febrile in comparison to afebrile laboring women (increased in expression in febrile to the right and increase in expression in afebrile to the left). Yellow color denotes significant P values, red dots with labels represent Bonferroni adjusted p values. **b.** Heatmap of spearman correlation of species only observed in V3-V4 (_V3-V4) or only V1-V2(_V1-V2) primers. Spearman correlation is between (-1,1). **c.** Scatter plot representing correlation between the *L. reuteri* (found only by V3-V4 primers) across samples and phascolarctobacterium (found only by V1-V2 primers) (correlation value = 0.73). **d.** Box plot of differentially expressed bacteria in febrile versus afebrile women on genus level (the ordinate represents log scale). **e,** Box plot of Levenshtein Distance for the sequence of various Streptococcus and Staphylococcus species. Different colors of box plot represent the 16s rRNA sequencing assays, each dot represents the Levenshtein distance value of two sequences.

Supplemental Fig. 5: V1-V2 topic model and Univariate Models for V3-V4

a. Cluster determination using nonnegative matrix factorization, a method for determining the appropriate number of clusters concordant with our dataset. CPI measure defines Cluster Purity Index, calculated as the percent of the total number of objects (data points) classified correctly in the unit range [0-1]). K denotes the number of clusters. **b.** V1-V2 omega value or the weight in which each sample is partitioned into either of the four clusters determined by the topic model. **c.** z score row-wise normalized. Bacterial weight across topics (theta) value of V1-V2 topic model for every feature (species taxonomic rank). **d**. Forest plot result of univariate GLM model for determination of unique feature with each topic. Abscissa denotes **β** value of GLM. Odds ratio is calculated as the exp(**β**) for binary variables.

Supplemental Fig. 6: Random Forest for V1-V2 and significant contributing features of maternal fever status determination

a. Density plot of calculated AUC for 100 models run with various test and training sets for our sample set using V3-V4 primers (similar plots were observed for V1-V2 min=0.55, max=0.9, mean=0.73). **b.** Feature importance identification for maternal fever status determination using RF model. **c.** Receiver operator curve (ROC) of V1-V2 random forest (RF) model for maternal fever status determination (all features versus clinical features or microbial features). **d.** Box plots and bar plot for the top six most important features determining maternal fever status in V1-V2 and V3-V4. Each dot denotes a sample in the continuous variable.

Supplemental Fig. 7: Relative Abundance Box Plots of Various Lactobacillus Species in Presence or Absence of Intrapartum Fever. X-axis represents the relative abundance of specific lactobacillus species/genera. Y-axis represents the maternal febrile status. Each dot is the agglomerated relative abundance of the particular species within the febrile or afebrile samples. Note the Y-axis for Enterobacter and Staphylococcus genera is log10.

Reference

1. Ravel,J., Gajer,P., Abdo,Z., Schneider,G.M., Koenig,S.S.K., McCulle,S.L., Karlebach,S., Gorle,R., Russell,J., Tacket,C.O., *et al.* (2011) Vaginal microbiome of reproductive-age women. *Proc. Natl. Acad. Sci. U. S. A.*, **108 Suppl 1**, 4680–4687.

2. Huang,B., Fettweis,J.M., Brooks,J.P., Jefferson,K.K. and Buck,G.A. (2014) The changing landscape of the vaginal microbiome. *Clin. Lab. Med.*, **34**, 747–761.

3. Bayigga,L., Kateete,D.P., Anderson,D.J., Sekikubo,M. and Nakanjako,D. (2019) Diversity of vaginal microbiota in sub-Saharan Africa and its effects on HIV transmission and prevention. *Am. J. Obstet. Gynecol.*, **220**, 155–166.

4. Bisanz,J.E., Enos,M.K., PrayGod,G., Seney,S., Macklaim,J.M., Chilton,S., Willner,D., Knight,R., Fusch,C., Fusch,G., *et al.* (2015) Microbiota at Multiple Body Sites during Pregnancy in a Rural Tanzanian Population and Effects of Moringa-Supplemented Probiotic Yogurt. *Appl. Environ. Microbiol.*, **81**, 4965–4975.

5. Graspeuntner,S., Loeper,N., Künzel,S., Baines,J.F. and Rupp,J. (2018) Selection of validated hypervariable regions is crucial in 16S-based microbiota studies of the female genital tract. *Sci. Rep.*, **8**, 9678.

6. Chakravorty,S., Helb,D., Burday,M., Connell,N. and Alland,D. (2007) A detailed analysis of 16S ribosomal RNA gene segments for the diagnosis of pathogenic bacteria. *J. Microbiol. Methods*, **69**, 330–339.

Supplemental Fig. 1

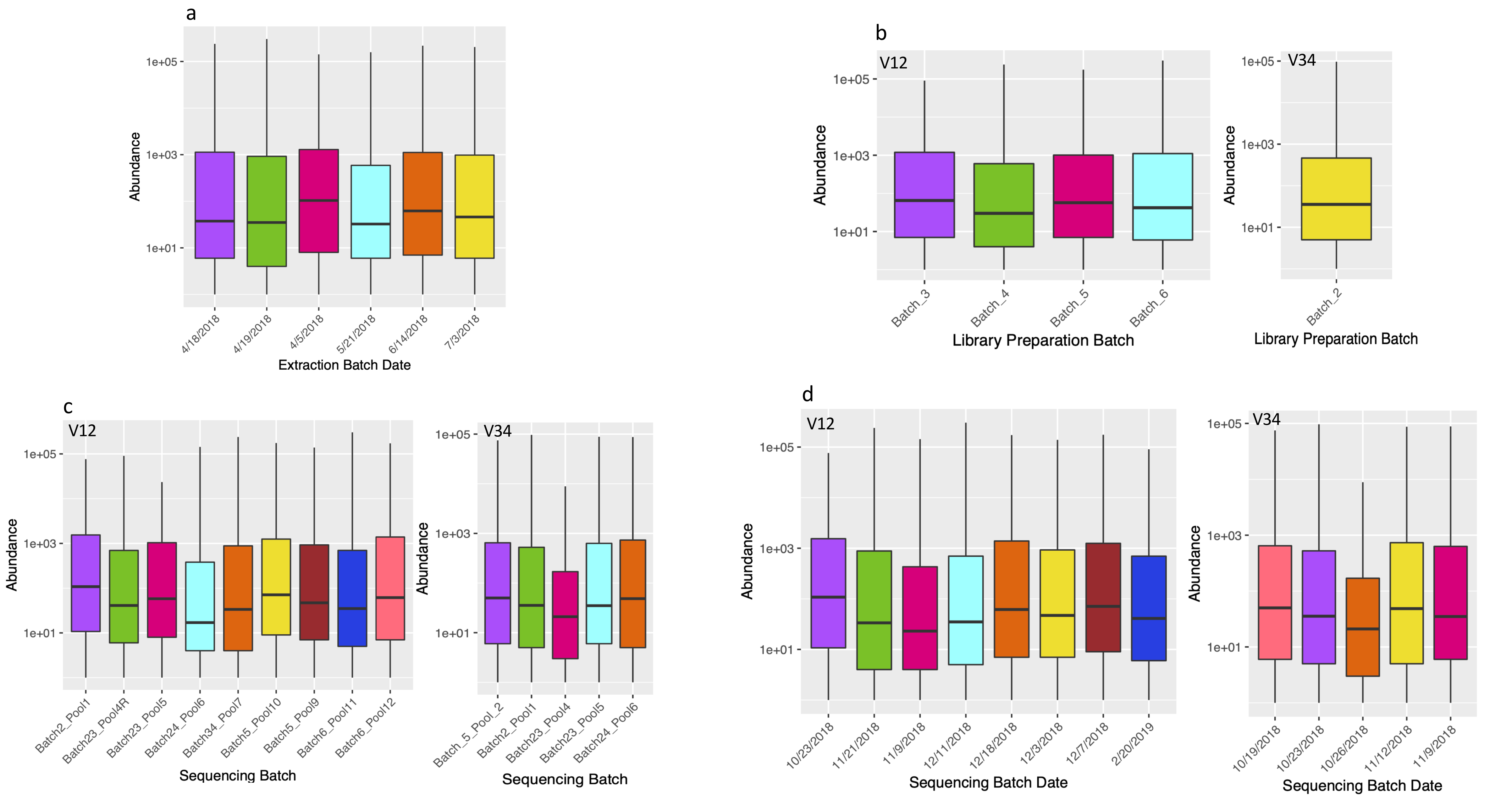

Supplemental Fig. 2

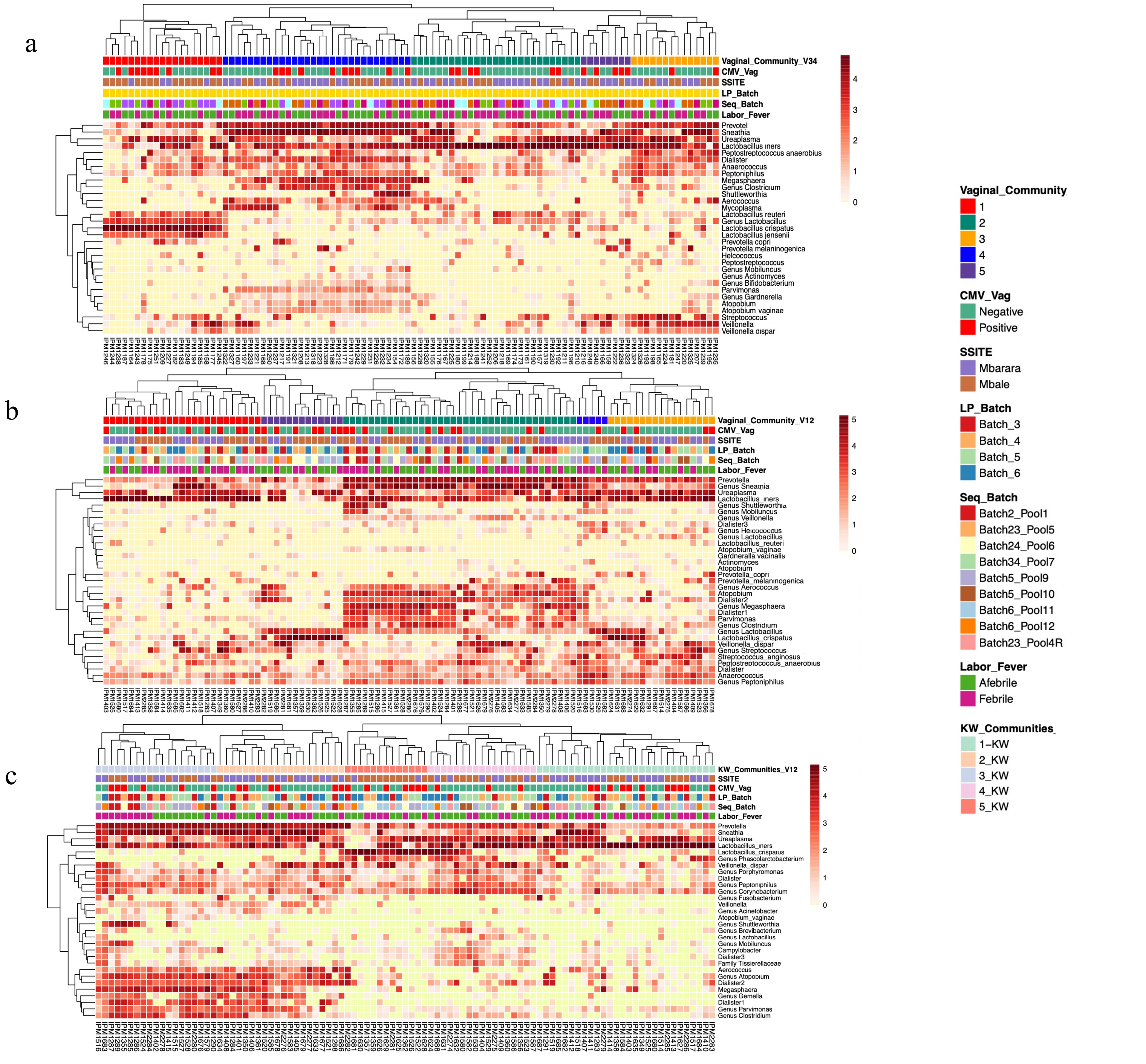

Supplemental Fig. 3

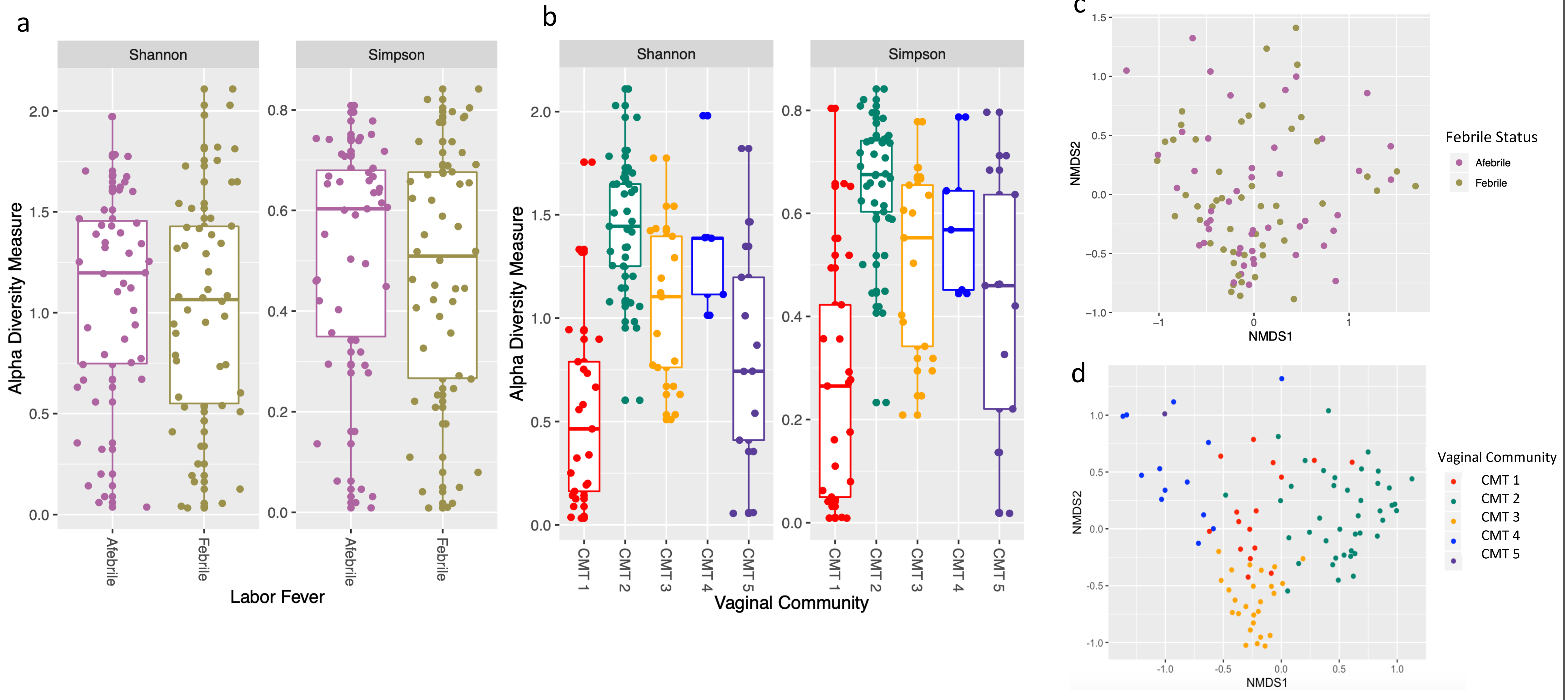

Supplemental Fig. 4

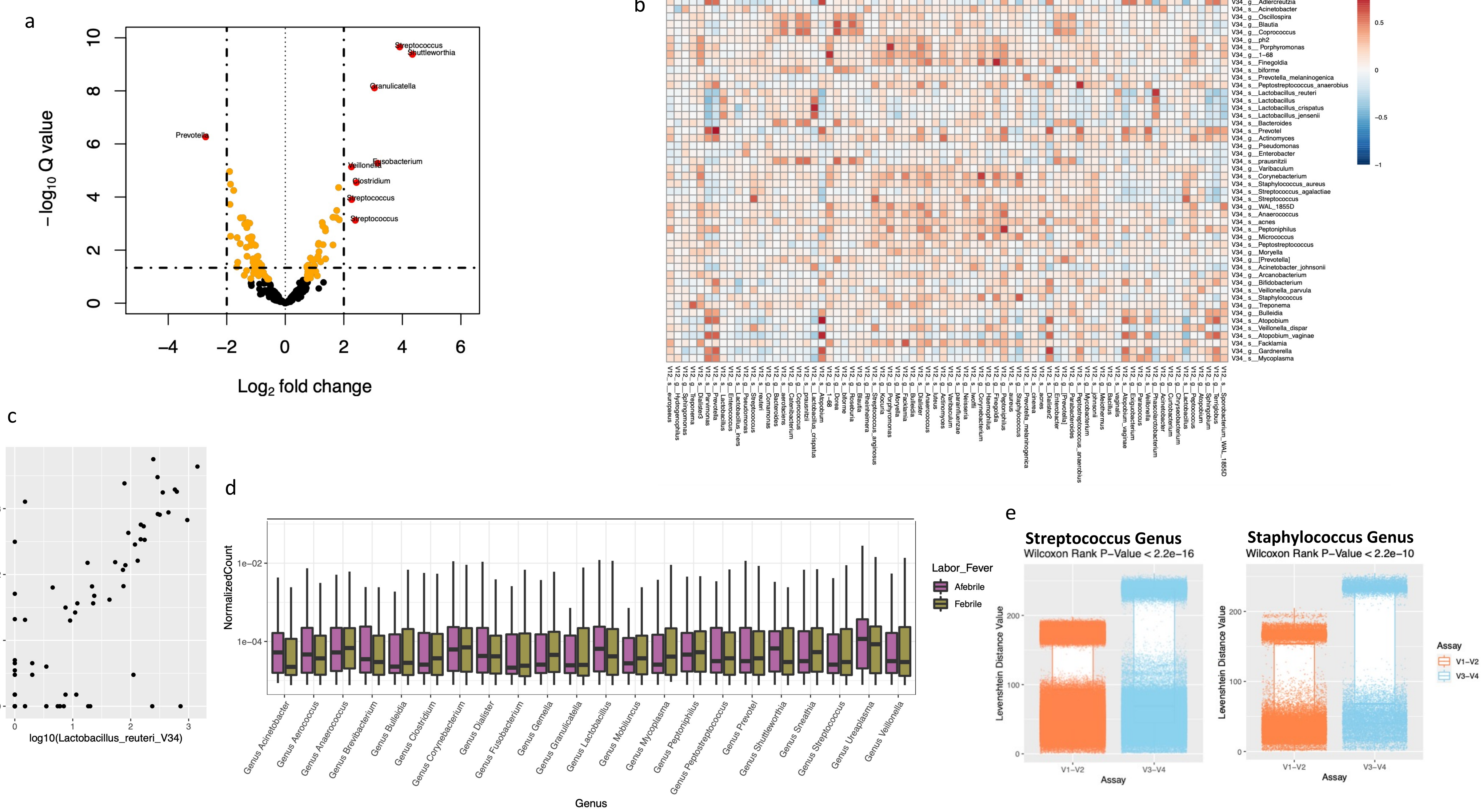

Supplemental Fig. 5

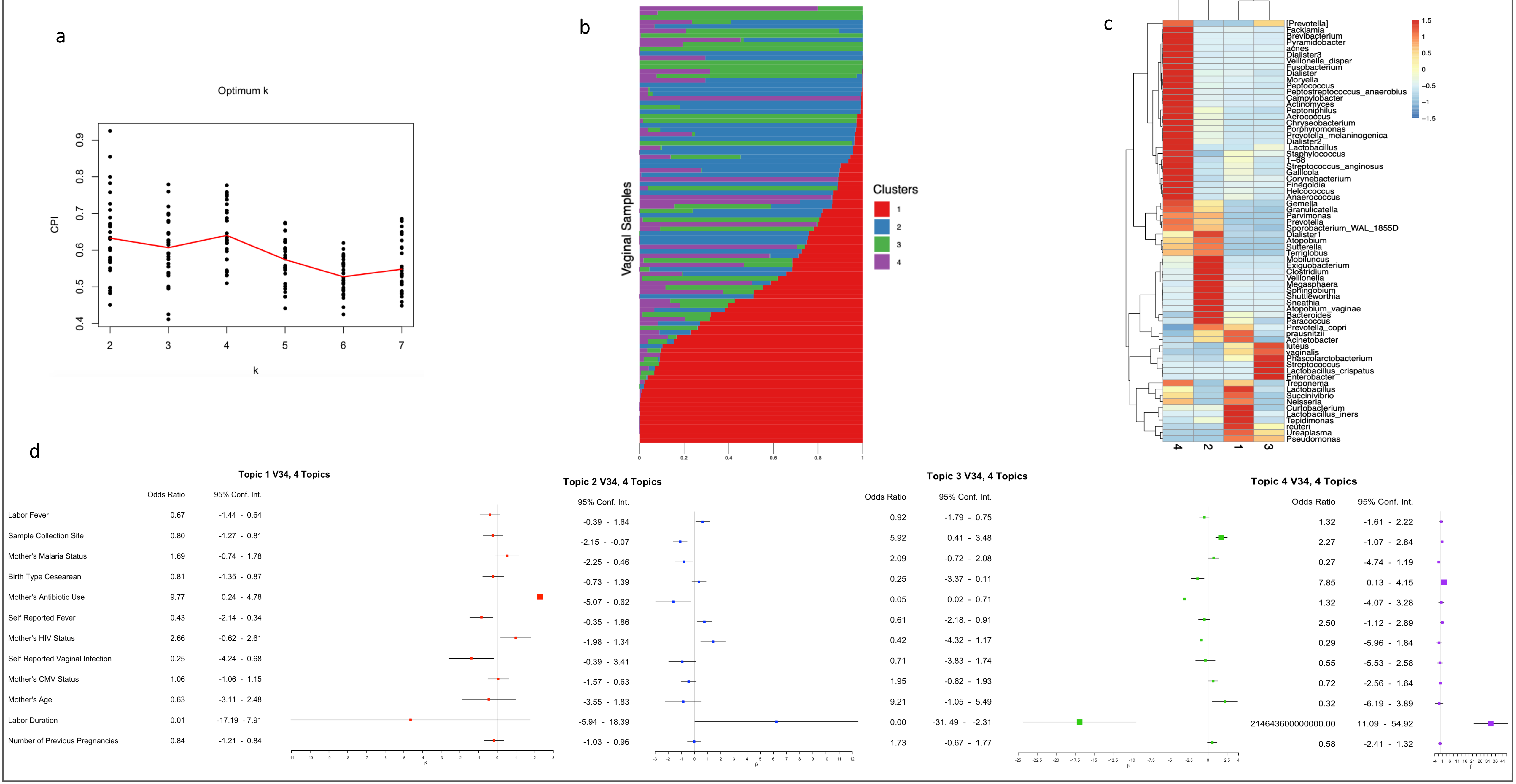

Supplemental Fig. 6

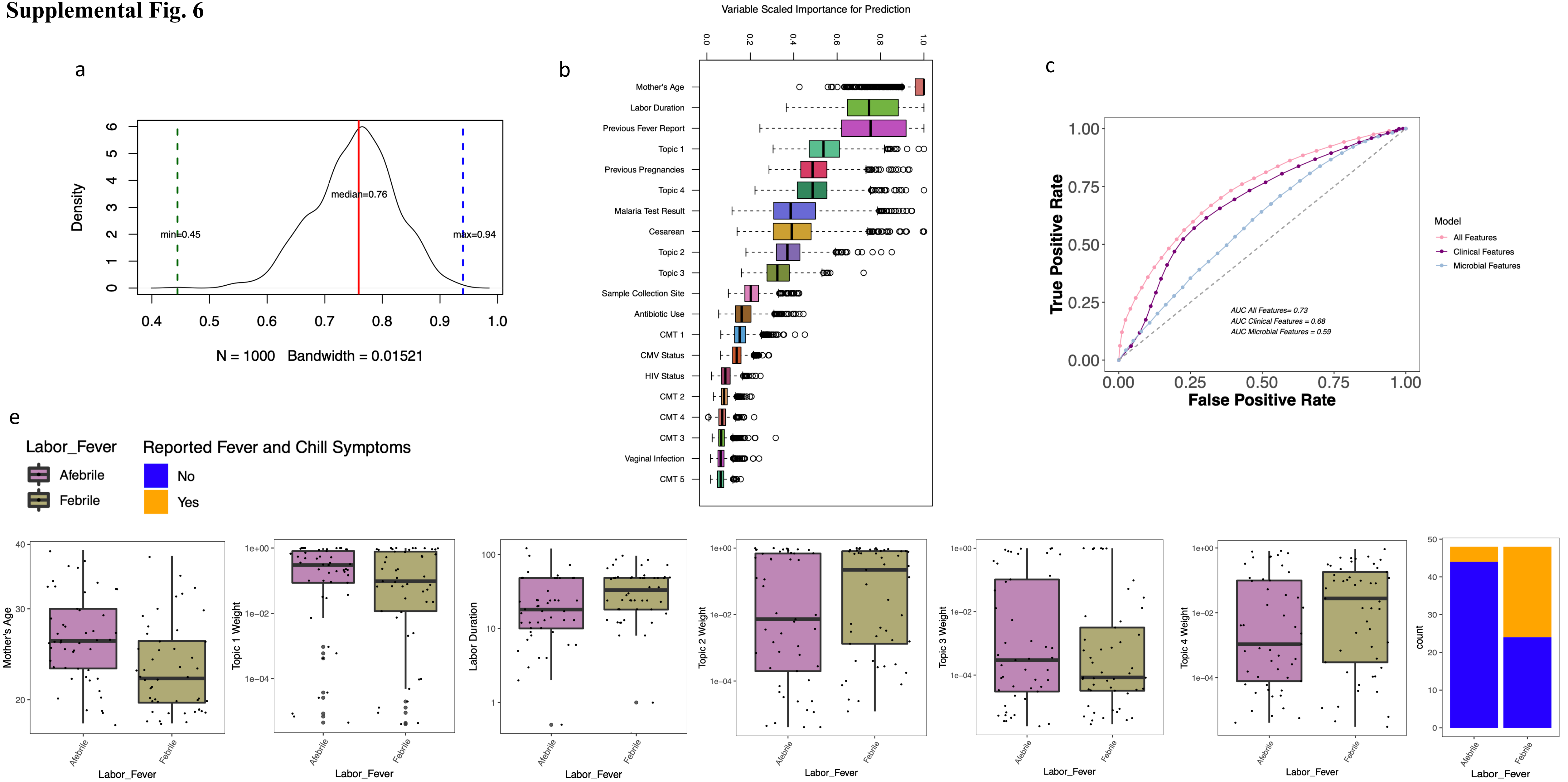

Supplemental Fig. 7

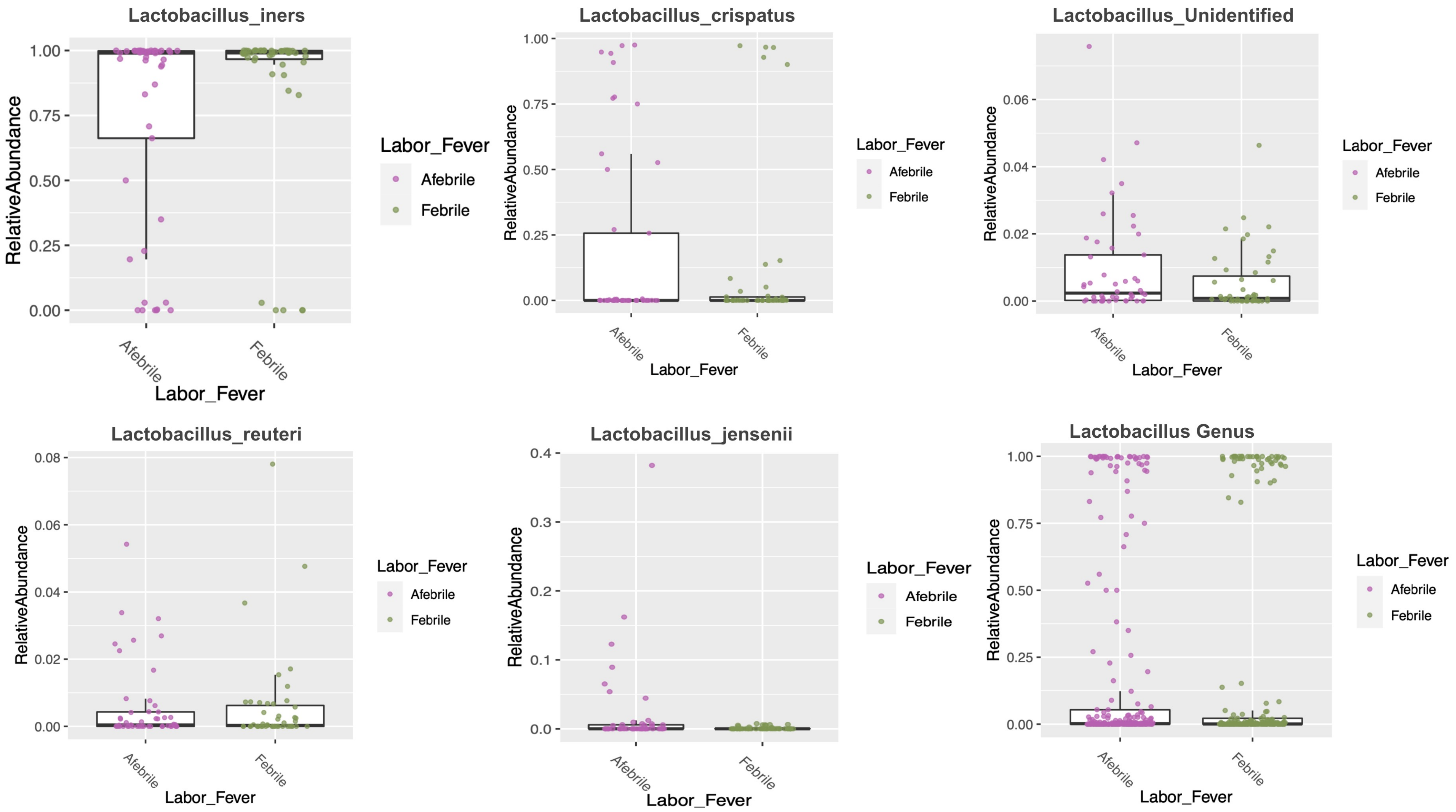

Supplementary Table 1: Full Overview clinical characterization febrile versus afebrile. a:N=(14,24),b:N=(26,16),c:N=(8,28),d:N=(48,49),e:N=(44,44),f:N=(8,28),g:N=(9,28)

Characteristic (N = 99)	*afebrile*	*febrile*	*Odds Ratio (95% CI)*
	n = 49	*n = 50*	Univariate
Occupation outside the home (Yes(%))	31(63%)	27(54%)	1.42(0.31-1.52)
Mean household income (UGx / K)(mean, SD) a	(400.769 , 304.170)	(348.125, 228.392)	0.99(-3.33,1.57)
Married	48(98%)	45(90%)	0.19(0.01-1.22)
Previous miscarriage or stillbirth (%)	5(10%)	5(10%)	0.98(0.26-3.42)
Antenatal clinic (ANC) attendance =4 versus >4 Yes(%)) b	33(67%)	21(43%)	0.35(6.58-0.79)
Self-reported urinary infection in last 1mo c	0(0%)	3(10%)	-
Number of vaginal exams in labor (median, IQR) d	(3,3)	(4,2)	1.07(0.94-1.26)
Gestational age at delivery, in weeks (median, SD) e	(40,1.2)	(40,1.2)	0.95(0.69-1.31)
Hospital diagnosis: sepsis f	2(33%)	1(3%)	0.11(0.01-1.34)
Hospital diagnosis: chorioamnionitis g	0(0%)	12(42%)	-

Supplementary Table 2: Concordance metrics assuming the microbiology bacteria recovery result data is the gold standard when compared to 16S rRNA gene V3-V4 and V1-V2 sequencing. FP denotes False positive rate, FN denotes False negative, FO False ommision

Measurement	Klebsiella V3-V4	Strep. Agalactiae V3-V4	Staph. Aureus V3-V4	Corynebacterium V3-V4	Klebsiella V1-V2	Corynebacterium V1-V2
Sensitivity/Recall	0.64	0	1	1	0	0.5
Specificity	0.8	0.65	0.79	0.06	1	0.81
Precision	0.28	0	0.05	0.03	NA	0.05
FP rate	0.2	0.35	0.21	0.93	0	0.19
FN rate	0.36	1	0	0	1	0.5
FO rate	0.72	1	0.79	0.97	NA	0.95

Supplementary Table 3: Kruskal wallis test results for bacteria significantly different across communities utilizing hierarchical clustering

Species	pvalue V3-V4	p_adj V3-V4	pvalue V1-V2	p_adj V1-V2
s__Atopobium_vaginae	6.19E-12	3.10E-10	7.29E-13	3.50E-11
g__Clostridium	5.80E-09	2.90E-07	5.60E-12	2.69E-10
s__Dialister1	NA	NA	2.63E-11	1.26E-09
s__Lactobacillus	3.16E-10	1.58E-08	7.49E-11	3.59E-09
s__Megasphaera	3.03E-12	1.51E-10	1.48E-10	7.12E-09
s__Lactobacillus_crispatus	5.87E-11	2.94E-09	1.61E-10	7.72E-09
s__Parvimonas	1.56E-12	7.79E-11	4.20E-10	2.01E-08
s__Prevotella	NA	NA	1.06E-09	5.11E-08
s__Atopobium	1.44E-11	7.22E-10	1.60E-09	7.70E-08
s__Veillonella	2.80E-05	1.40E-03	2.46E-09	1.18E-07
g__Gemella	4.69E-10	2.35E-08	4.37E-08	2.10E-06
s__Dialister2	NA	NA	7.21E-08	3.46E-06
s__Sneathia	4.98E-10	2.49E-08	8.44E-08	4.05E-06
s__Lactobacillus_iners	4.59E-10	2.29E-08	1.51E-07	7.27E-06
g__Phascolarctobacterium	NA	NA	1.62E-07	7.78E-06
s__Veillonella_dispar	1.46E-05	7.31E-04	1.16E-06	5.56E-05
s__Aerococcus	1.20E-09	6.00E-08	1.76E-06	8.45E-05
g__Brevibacterium	NA	NA	8.08E-06	3.88E-04
s__ Porphyromonas	1.15E-01	1.00E+00	1.18E-05	5.65E-04
s__Peptoniphilus	1.24E-03	6.21E-02	1.19E-05	5.69E-04
s__Campylobacter	NA	NA	4.33E-05	2.08E-03
f__[Tissierellaceae]_g__1−6	2.14E-02	1.00E+00	1.62E-04	7.76E-03
s__Shuttleworthia	7.76E-02	1.00E+00	1.77E-04	8.49E-03
s__Dialister	1.66E-09	8.32E-08	1.77E-04	8.50E-03
s__Acinetobacter	2.93E-02	1.00E+00	2.18E-04	1.05E-02
s__Dialister3	NA	NA	5.12E-04	2.46E-02
g__Mobiluncus	1.76E-01	1.00E+00	5.36E-04	2.57E-02
s__Fusobacterium	6.43E-05	3.21E-03	8.58E-04	4.12E-02
s__Ureaplasma	6.44E-03	3.22E-01	1.18E-03	5.67E-02

s__Corynebacterium	1.25E-03	6.24E-02	1.26E-03	6.04E-02
s__Staphylococcus	3.77E-04	1.88E-02	3.26E-03	1.56E-01
g__Gardnerella	5.57E-06	2.78E-04	3.29E-03	1.58E-01
s__Prevotella_melaninogenic	1.02E-02	5.10E-01	3.77E-03	1.81E-01
s__Prevotella_copri	1.30E-02	6.48E-01	4.70E-03	2.26E-01
g__Pseudomonas	NA	NA	5.00E-03	2.40E-01
s__Peptostreptococcus_anaer	1.63E-03	8.17E-02	7.56E-03	3.63E-01
s__Helcococcus	4.91E-02	1.00E+00	8.32E-03	4.00E-01
s__Anaerococcus	2.10E-02	1.00E+00	1.10E-02	5.30E-01
s__Finegoldia	1.63E-04	8.13E-03	1.16E-02	5.56E-01
g__Enterobacter	2.23E-01	1.00E+00	6.41E-01	1.00E+00
s__Granulicatella	6.31E-04	3.16E-02	8.77E-02	1.00E+00
s__Lactobacillus_reuteri	1.78E-08	8.90E-07	3.32E-01	1.00E+00
s__Streptococcus	1.82E-04	9.11E-03	4.50E-02	1.00E+00
g__Bacteroides	NA	NA	9.80E-01	1.00E+00
s__Streptococcus_anginosus	NA	NA	4.37E-02	1.00E+00
g__Actinomyces	2.46E-05	1.23E-03	NA	NA
g__Adlercreutzia	6.42E-12	3.21E-10	NA	NA
g__Bifidobacterium	1.87E-06	9.37E-05	NA	NA
g__Brevibacterium	3.17E-01	1.00E+00	NA	NA
g__Bulleidia	7.64E-06	3.82E-04	NA	NA
g__Campylobacter	2.37E-02	1.00E+00	NA	NA
g__Coprococcus	3.10E-01	1.00E+00	NA	NA
g__Micrococcus	1.22E-03	6.08E-02	NA	NA
g__Moryella	5.20E-02	1.00E+00	NA	NA
g__ph2	4.26E-02	1.00E+00	NA	NA
s__Facklamia	2.55E-01	1.00E+00	NA	NA
s__Lactobacillus_jensenii	2.59E-11	1.29E-09	NA	NA
s__Mycoplasma	6.43E-08	3.22E-06	NA	NA
s__Peptostreptococcus	7.36E-04	3.68E-02	NA	NA
s__Prevotel	4.20E-10	2.10E-08	NA	NA

Supplementary Table 4: Bacterial presence or absence in each community versus topic . >= 75% quartile of the samples retain bacteria in the community is shown in pink, 2nd to 3rd quartile of samples retaining the bacteria in the community is shown in yellow and =< 25 % quarter of samples is purple (Note Overall the table shows presence or absence of the bacteria in the community and does not incorportate levels, white signifies not present, light blue reprsents not found to be different in comminities using Kruskal Wallis test). A similar logic was used for soecific topics in the GoM models with the difference of relying on the weight of every bacteria in the each topic (shown as theta levels-high versus medium versus low, in the theta plot when high the heatmap value >0.5, if med -0.5<theta value <0.5, and for low <-0.5).

Key:	Not present	Not associated	Low	Medium	High

Species	Community 1	Community 2	Community 3	Community 4a	Community 4b	Topic 1	Topic 2	Topic 3	Topic 4
Acinetobacter	Not present	Low	Not present	Medium	Low	Medium	High	Medium	Low
Aerococcus	Low	Medium	Medium	High	High	Low	Medium	Low	High
Anaerococcus	Not associated	Not associated	Not associated	Not associated	Not associated	Low	Low	Medium	High
Atopobium	Low	Low	Not present	High	High	Low	High	Low	Medium
Atopobium_vaginae	Low	Low	Not present	High	High	Low	High	Low	Medium
Corynebacterium	High	High	High	High	High	Low	Low	High	Medium
Dialister	Medium	Medium	High	High	High	Low	High	Low	High
Enterobacter	Not associated	Not associated	Not associated	Not associated	Not associated	High	Low	Medium	Low
Facklamia	Not associated	Not associated	Not associated	Not associated	Not associated	Low	Low	High	Medium
Finegoldia	Not associated	Not associated	Not associated	Not associated	Not associated	Low	Low	Medium	High
Fusobacterium	Low	Low	Medium	Low	Medium	Low	Low	Low	High
Granulicatella	Low	Medium	Medium	Low	Medium	Low	Low	Low	High
Helcococcus	Not associated	Not associated	Not associated	Not associated	Not associated	Low	Low	High	Low
Lactobacillus	High	Medium	Medium	Medium	Medium	Low	Low	High	Low
Lactobacillus_crispatus	High	Medium	Low	Low	Low	Low	Low	High	Low
Lactobacillus_iners	High	High	High	High	High	Low	Low	High	Low
Lactobacillus_jensenii	High	Medium	Low	Not present	Low	Low	Low	High	Low
Lactobacillus_reuteri	High	Medium	Medium	Low	Medium	Medium	Low	High	Low
Megasphaera	Low	Medium	Medium	High	Medium	High	Low	Low	Low
Mycoplasma	Low	Low	Not present	Medium	Medium	High	Low	Low	Low
Parvimonas	Low	Low	Low	High	Medium	High	Low	Low	Low
Peptoniphilus	High	High	High	High	High	Low	High	Low	High

Peptostreptococcus_anaerobius									
Peptostreptococcus									
Porphyromonas									
Prevotel									
Prevotella_copri									
Prevotella_melaninogenica									
Shuttleworthia									
Sneathia									
Staphylococcus									
Streptococcus									
Ureaplasma									
Veillonella									
Veillonella_dispar									

Supplementary Table 5: Multivariate Regression of various data features in relation to each topic. T(1-4) stands for Topic (1-4), BC P Value represents bonferroni corrected P value red represents signficant P values (adj P <0.05)

Feature	Beta T1	Std.Error T1	P Value T1	BC P Value T1
Labor Fever	-0.12094	0.10679	0.2613	1
SSITE2	-0.15578	0.10264	0.1337	1
PREGN Group 1 and 2	0.02259	0.03097	0.4682	1
Malaria test result	0.24125	0.11478	0.0392	0.55
HIV status	0.19198	0.14236	0.1819	1
Mother's Age Group <26 and 26=>	-0.11908	0.12532	0.3453	1
Vaginal infection report	-0.36494	0.16707	0.0323	0.45
Labor duration Group 12hrs	-0.10349	0.11428	0.3683	1
Labor duration Group 24_48hrs	-0.05917	0.1227	0.6312	1
Labor duration Group 48_Above	-0.16971	0.12096	0.1651	1
Previous chills and fever reported	-0.07916	0.12364	0.5241	1
CMV	-0.06536	0.09655	0.5007	1
Antibiotic Use	0.6054	0.19118	0.0023	0.03
Ceseasrean	-0.0101	0.09847	0.9186	1

Feature	Beta T2	Std.Error T2	P Value T2	BC P Value T2
Labor Fever	0.161207	0.117259	0.1736	1
SSITE2	-0.109584	0.112706	0.3343	1
PREGN Group 1 and 2	-0.018432	0.034002	0.5895	1
Malaria test result	-0.22175	0.126033	0.0829	1
HIV status	-0.050122	0.15632	0.7495	1
Mother's Age Group <26 and 26=>	0.01266	0.13761	0.927	1
Vaginal infection report	0.30752	0.183451	0.0982	1
Labor duration Group 12hrs	-0.024944	0.12548	0.843	1
Labor duration Group 24_48hrs	-0.009136	0.134733	0.9461	1

Labor duration Group 48_Above	0.142532	0.132819	0.2869	1
Previous chills and fever reported	-0.097779	0.135757	0.4738	1
CMV	0.094388	0.10601	0.3764	1
Antibiotic Use	-0.336014	0.209925	0.114	1
Ceseasrean	0.022402	0.10812	0.8365	1

Feature	Beta T3	Std.Error T3	P Value T3	BC P Value T3
Labor Fever	-0.013042	0.100391	0.897	1
SSITE2	0.15261	0.096493	0.118	1
PREGN Group 1 and 2	-0.009626	0.02911	0.742	1
Malaria test result	0.021536	0.107902	0.842	1
HIV status	-0.049628	0.133832	0.712	1
Mother's Age Group <26 and 26=>	0.113459	0.117813	0.339	1
Vaginal infection report	0.049054	0.15706	0.756	1
Labor duration Group 12hrs	0.065989	0.107429	0.541	1
Labor duration Group 24_48hrs	-0.03739	0.115351	0.747	1
Labor duration Group 48_Above	-0.017914	0.113712	0.875	1
Previous chills and fever reported	0.084211	0.116228	0.471	1
CMV	0.02688	0.090759	0.768	1
Antibiotic Use	-0.197938	0.179725	0.275	1
Ceseasrean	-0.079966	0.092566	0.391	1

Feature	Beta T4	Std.Error T4	P Value T4	BC P Value T4
Labor Fever	-0.027225	0.063978	0.672	1
SSITE2	0.11275	0.061494	0.071	0.99
PREGN Group 1 and 2	0.005469	0.018552	0.769	1
Malaria test result	-0.041033	0.068765	0.553	1
HIV status	-0.092233	0.08529	0.283	1

Mother's Age Group <26 and 26=>	-0.007039	0.075081	0.926	1
Vaginal infection report	0.008366	0.100093	0.934	1
Labor duration Group 12hrs	0.062449	0.068463	0.365	1
Labor duration Group 24_48hrs	0.105692	0.073512	0.155	1
Labor duration Group 48_Above	0.045087	0.072467	0.536	1
Previous chills and fever reported	0.092729	0.07407	0.215	1
CMV	-0.055911	0.05784	0.337	1
Antibiotic Use	-0.07145	0.114537	0.535	1
Ceseasrean	0.067668	0.058991	0.255	1

Supplementary Table 6: Presence and absence table for Lactobacillus Species. Note: F stands for Febrile and A stands for Afebrile										
Lactobacillus Species	# Samples Positive CMT1 - F	# Samples Positive CMT2 - F	# Samples Positive CMT3 - F	# Samples Positive CMT4 - F	# Samples Positive CMT5 - F	# Samples Positive CMT1 - A	# Samples Positive CMT2 - A	# Samples Positive CMT3 - A	# Samples Positive CMT4 - A	# Samples Positive CMT5 - A
Lactobacillus_iners	7	18	5	10	8	11	18	2	6	10
Lactobacillus_crispatus	7	12	2	5	2	12	8	2	2	2
Lactobacillus_jensenii	7	10	2	4	0	12	12	0	1	0
Lactobacillus_reuteri	6	12	2	4	1	11	14	1	3	0
Lactobacillus_Unidentifie[illegible]	7	9	2	5	6	12	9	2	5	9

Supplementary Table 7: Table for list of all primers used for the paper (5'-3')

Organism	Primer Name	Sequence
CMV (Human Herpes Virus 5)	CPOL-F720	GCTGACGCGTTTGGTCATC
	CPOL-R780	ACGATT CAC GGA GCA CCAG
	CPOL-741FAM	TCGGCGGATCACCACGTT CG
Hypervariable regions 1, 2 and 3, 4	27F	AGAGTTTGATCMTGGCTCAG
	M13	CAGGGTTTTCCCAGTCACGAC
	341F_M13	CAGGGTTTTCCCAGTCACGACCCTACGGGNGGCWGCAG
	805R	GACTACHVGGGTATCTAATCC
