# Supplementary material for: Vaginal microbiome topic modeling of laboring Ugandan women with and without fever
Source: NPJ Biofilms Microbiomes. 2021 Sep 10;7:75. doi: 10.1038/s41522-021-00244-1 (PMC8433417; doi:10.1038/s41522-021-00244-1)
Supplement: Supplementary file 2 — Reporting Summary [file 41522_2021_244_MOESM2_ESM.pdf]

nature research

Corresponding author(s): Joseph N. Paulson

Last updated by author(s): Jun 4, 2021

Reporting Summary

Nature Research wishes to improve the reproducibility of the work that we publish. This form provides structure for consistency and transparency in reporting. For further information on Nature Research policies, see our Editorial Policies and the Editorial Policy Checklist.

Statistics

For all statistical analyses, confirm that the following items are present in the figure legend, table legend, main text, or Methods section.

n/a	Confirmed	
☐	☒	The exact sample size (*n*) for each experimental group/condition, given as a discrete number and unit of measurement
☐	☒	A statement on whether measurements were taken from distinct samples or whether the same sample was measured repeatedly
☐	☒	The statistical test(s) used AND whether they are one- or two-sided<br>*Only common tests should be described solely by name; describe more complex techniques in the Methods section.*
☐	☒	A description of all covariates tested
☐	☒	A description of any assumptions or corrections, such as tests of normality and adjustment for multiple comparisons
☐	☒	A full description of the statistical parameters including central tendency (e.g. means) or other basic estimates (e.g. regression coefficient) AND variation (e.g. standard deviation) or associated estimates of uncertainty (e.g. confidence intervals)
☐	☒	For null hypothesis testing, the test statistic (e.g. *F*, *t*, *r*) with confidence intervals, effect sizes, degrees of freedom and *P* value noted<br>*Give P values as exact values whenever suitable.*
☐	☒	For Bayesian analysis, information on the choice of priors and Markov chain Monte Carlo settings
☐	☒	For hierarchical and complex designs, identification of the appropriate level for tests and full reporting of outcomes
☐	☒	Estimates of effect sizes (e.g. Cohen's *d*, Pearson's *r*), indicating how they were calculated

Our web collection on statistics for biologists contains articles on many of the points above.

Software and code

Policy information about availability of computer code

Data collection	No specialized code was used for data collection.
Data analysis	Sequence alignment and taxonomic assignment<br>Paired-end sequences were filtered for quality using Trimmomatic (version (v) 0.38)(64). Sequences <100 base pairs (bp) in length and average quality score <30 on a window of 20 bps were discarded. The remaining paired-end sequences were then joined utilizing PEAR v 0.9.6) (65). Only joined sequences with designed primers and length > 260 bp were kept. After chimeras were identified and removed using USEARCH method (66), sequences were clustered into operational taxonomic units (OTUs) via QIIME packages (v 1.9.1) (67). Sequences of over97 percent identity represented the same genus/species, and were clustered into the same OTU, and were assigned a taxonomy by Greengenes database (v 13.8). Those OTUs without taxonomy assignment were further blasted with BlastX (v 2.7.1) to non-redundant proteins (NR) databases.<br>Computational pipelines and statistical methods for downstream analysis<br>The biological observation matrix (BIOM) object compromising operational taxonomic unit (OTU) file, phenotype data and taxonomic assignment file was built using phyloseq and metagenomeSeq packages in the R programming language (68, 69). Minimum inclusion criteria for sequenced samples was 1000 reads. Taxa with less than 2 reads in 10% of samples were excluded from the analysis. The number of Operative Taxonomic Units (OTUs) after filtering using V1-V2 primers was 274 and utilizing V3-V4 primers was 401 OTUs. For the hypervariable region/primer comparison section, we relaxed the filtering criteria to taxa with fewer than 1 reads in 5% samples to ensure accurate estimates of presence and absence association tests for bacteria across both regions.<br>Packages used for computational analysis<br>Beta diversity was estimated using non-metric multidimensional scaling (NMDS). Euclidean distance was measured for hierarchical clustering using the pheatmap package (v 1.0.12). Phyloseq and ggplot2 were used to estimate alpha (Shannon and Simpson) and beta (non-metric multidimensional scaling) diversity and visualization of plots accordingly (68, 70). Both adjusted and unadjusted differential abundance analyses were performed using DESeq2 (71). Multivariate models were adjusted for microbial community and site of collection. GoM models identified were obtained using the CountClust package(72). Univariate and multivariate regressions were performed for topic weights

regressing on various clinical features depending on the type of regression and adjusted by the number of models (i.e. based on the number of topics). Random forest (RF) models were utilized for maternal fever status prediction from the h2o package (v 3.30.0.1, https://github.com/h2oai/h2o-3).
Statistical tests
All statistical tests and regression analyses were performed using R base functions or MASS (73). All P values were adjusted using Bonferroni multiple comparison test method R v 3.6.2.

For manuscripts utilizing custom algorithms or software that are central to the research but not yet described in published literature, software must be made available to editors and reviewers. We strongly encourage code deposition in a community repository (e.g. GitHub). See the Nature Research guidelines for submitting code & software for further information.

Data

Policy information about availability of data

All manuscripts must include a data availability statement. This statement should provide the following information, where applicable:
- Accession codes, unique identifiers, or web links for publicly available datasets
- A list of figures that have associated raw data
- A description of any restrictions on data availability

The 16S rRNA sequencing files and metadata can be accessed through https://microbiomedb.org/ as well as NCBI SRA under BioProject accession #PRJNA672786. All scripts and statistical methodology can be found in https://github.com/DataScienceGenomics/Vaginal_Microbiome.git.

Field-specific reporting

Please select the one below that is the best fit for your research. If you are not sure, read the appropriate sections before making your selection.

☒ Life sciences ☐ Behavioural & social sciences ☐ Ecological, evolutionary & environmental sciences

For a reference copy of the document with all sections, see nature.com/documents/nr-reporting-summary-flat.pdf

Life sciences study design

All studies must disclose on these points even when the disclosure is negative.

Sample size	The study was an observational cohort of 100 patients. Biomarker samples were processed for those with available samples.
Data exclusions	For OTU exclusion / removal in comparing hypervariable regions / primers we relaxed the filtering criteria to taxa with fewer than 1 reads in 5% samples to ensure accurate estimates of presence and absence association tests for bacteria across both regions.
Replication	Not applicable
Randomization	Not applicable
Blinding	Not applicable

Reporting for specific materials, systems and methods

We require information from authors about some types of materials, experimental systems and methods used in many studies. Here, indicate whether each material, system or method listed is relevant to your study. If you are not sure if a list item applies to your research, read the appropriate section before selecting a response.

Materials & experimental systems

n/a	Involved in the study	
☒	☐	Antibodies
☒	☐	Eukaryotic cell lines
☒	☐	Palaeontology and archaeology
☒	☐	Animals and other organisms
☐	☒	Human research participants
☒	☐	Clinical data
☒	☐	Dual use research of concern

Methods

n/a	Involved in the study	
☒	☐	ChIP-seq
☒	☐	Flow cytometry
☒	☐	MRI-based neuroimaging

Human research participants

Policy information about studies involving human research participants

Population characteristics	99 laboring Ugandan women with and without intrapartum fever with term pregnancies. Women presenting to either hospital in labor for delivery were eligible for enrollment if they were aged 18 years or older, delivered at term (≥37 weeks'

	gestation), and had an intrapartum oral temperature measurement between 36.0-37.5 °C (afebrile group, n=25 per site); or a in intrapartum oral temperature measurement >38.1 °C on one occasion or >38.0 °C twice, at least 60 minutes apart (febrile group, n=25 per site).
Recruitment	An equal number of participants were recruited from Mbarara Regional Referral Hospital (MbararaH) in Mbarara, Uganda and Mbale Regional Referral Hospital in Mbale, Uganda (MbaleH). MbararaH is an approximately 300-bed academic hospital affiliated with Mbarara University of Science and Technology with 9,000 deliveries annually. MbaleH is a 400-bed public hospital that has nearly 10,000 deliveries a year. Both hospitals serve a mixed urban-agrarian population.
Ethics oversight	This study protocol was approved by the institutional review boards at each participating institution, including Mbarara University of Science and Technology (MUST) Research Ethics Committee (12/11-15), Mbale Regional Referral Hospital Research Ethics Committee (082/2016), Uganda National Council of Science and Technology (HS/1963), Partners (2016P000806/PHS), and Pennsylvania State University College of Medicine (STUDY0004199).

Note that full information on the approval of the study protocol must also be provided in the manuscript.
